# A semiparametric quantile regression rank score test for zero-inflated data

**DOI:** 10.1093/biomtc/ujaf050

**Published:** 2025-05-05

**Authors:** Zirui Wang, Wodan Ling, Tianying Wang

**Affiliations:** Department of Statistics and Data Science, Tsinghua University, Beijing, 100084, China; Division of Biostatistics, Department of Population Health Sciences, Weill Cornell Medicine, New York, NY 10065, USA; Department of Statistics, Colorado State University, Fort Collins, CO 80523, USA

**Keywords:** quantile regression, semi-parametric modeling, two-part model

## Abstract

Zero-inflated data commonly arise in various fields, including economics, healthcare, and environmental sciences, where measurements frequently include an excess of zeros due to structural or sampling mechanisms. Traditional approaches, such as Zero-Inflated Poisson and Zero-Inflated Negative Binomial models, have been widely used to handle excess zeros in count data, but they rely on strong parametric assumptions that may not hold in complex real-world applications. In this paper, we propose a zero-inflated quantile single-index rank-score-based test (ZIQ-SIR) to detect associations between zero-inflated outcomes and covariates, particularly when nonlinear relationships are present. ZIQ-SIR offers a flexible, semi-parametric approach that accounts for the zero-inflated nature of the data and avoids the restrictive assumptions of traditional parametric models. Through simulations, we show that ZIQ-SIR outperforms existing methods by achieving higher power and better Type I error control, owing to its flexibility in modeling zero-inflated and overdispersed data. We apply our method to the real-world dataset: microbiome abundance from the Columbian Gut study. In this application, ZIQ-SIR identifies more significant associations than alternative approaches, while maintaining accurate type I error control.

## 1 INTRODUCTION

Zero-inflated data arise in many fields, including economics (Lewsey and Thomson, 2004), healthcare (Wang et al., 2015), and environmental sciences (Agarwal et al., 2002), where excess zeros result from structural or sampling mechanisms. These zeros may indicate true absences or undetected values due to measurement limitations, complicating statistical analyses of response-covariate relationships. Traditional methods, such as Zero-Inflated Poisson (ZIP) and Zero-Inflated Negative Binomial (ZINB) models, are widely used but rely on strong parametric assumptions that often fail in real-world applications (Horton et al., 2007). Violations of these assumptions can inflate type I errors, particularly when covariates exhibit nonlinear relationships with the response (Hawinkel et al., 2019).

To relax the parametric assumptions in ZIP and ZINB, Ling et al. (2021b) proposed the zero-inflated quantile rank-score-based test (ZIQRank), which mitigates strong assumptions on non-zero counts by incorporating ordinary quantile regression (Koenker and Bassett, 1978). ZIQRank has been shown to achieve comparable or higher power than existing tests while better controlling false positives (Ling et al., 2021b). Unlike parametric methods, quantile regression is a robust tool for modeling complex data without distributional assumptions. However, its assumption on linear models at each quantile level may fail to capture complex relationships between zero-inflated outcomes and multivariate covariates, potentially leading to uncontrolled type I error inflation, particularly in sparse data or small samples. A similar issue has been reported in linear regression tests (Schwarzer et al., 2002).

**FIGURE 1:**
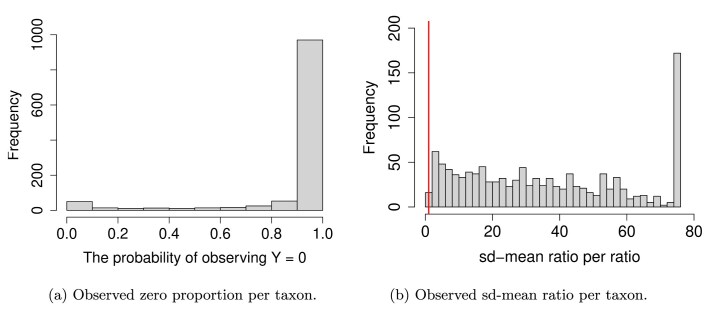
Histogram for Columbian’s gut dataset.

To highlight the limitations of ZIQRank, we present a motivating example from microbiome association studies. Human microbiomes play a crucial role in health and disease (Kinder-Haake, 2012), with alterations linked to conditions such as inflammatory bowel disease (Frank et al., 2007), cancer (Kostic et al., 2013), and major depressive disorder (Jiang et al., 2015). However, inflated zeros in microbial abundance and their complex relationships with biomedical factors pose significant challenges in analysis (Martin et al., 2020). To illustrate these complexities, we analyze a widely studied dataset from the Columbian Gut study (De la Cuesta-Zuluaga et al., 2018; Gonzalez et al., 2018), which includes 441 samples with gut microbiota profiled and health-related measures such as BMI and blood pressure (Kinder-Haake, 2012). Figure 1(a) reveals a high prevalence of zeros in microbial abundance, while Figure 1(b) shows that over 99% of taxa have a standard deviation-to-mean ratio greater than 1, indicating the overdispersion characteristic of microbiome data.

**FIGURE 2:**
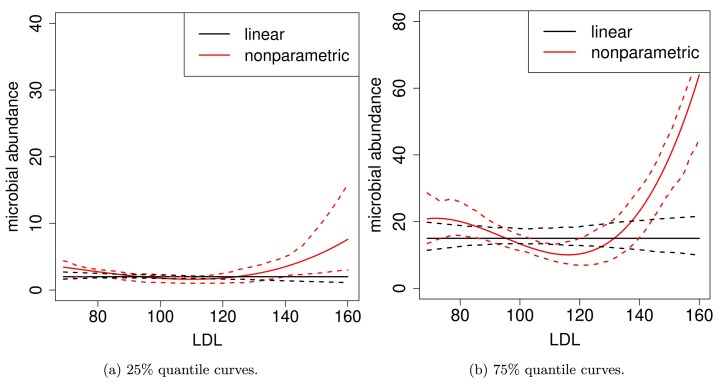
B-spline estimated 25% and 75% of quantile curves for taxon *Enterobacteriaceae*-unspecified fitted against the covariate LDL. The 90% confidence intervals (dashed lines) of the fitted quantile curves (solid lines) are constructed by bootstrap.

To illustrate the nonlinear relationship between zero-inflated outcomes and covariates, we examine the taxon *Enterobacteriaceae*, a key microbial family in the human body (De la Cuesta-Zuluaga et al., 2018), and model its abundance against low-density lipoprotein (LDL), which has been linked to *Enterobacteriaceae* levels (Liu et al., 2018). Since we focus on the relationship between positive taxon counts and covariates, zeros were excluded. We estimated the 25% and 75% quantile curves of non-zero taxon counts using both a B-spline-based nonparametric method (Eilers and Marx, 1996) and a linear quantile regression model (Koenker et al., 1994), the latter applied in Ling et al. (2021a). Bootstrap was used to construct 90% confidence intervals (Efron and Tibshirani, 1994). At both quantile levels, the linear quantile regression model detected no abundance changes with respect to LDL, whereas the nonparametric model indicated that high LDL levels are associated with increased *Enterobacteriaceae* abundance (Figure 2).

To detect associations between zero-inflated responses and covariates in the presence of non-linearity, we propose the zero-inflated quantile single-index rank-score-based test (ZIQ-SIR). Single-index models are widely used for handling complex data while ensuring interpretability (Neykov et al., 2016), making them suitable for various applications. This model assumes that covariates ${\bf X}$ influence the response $Y$ through a link function $G({\bf X}^\top \boldsymbol \beta )$, where $\boldsymbol \beta$ is an unknown parameter and $G$ is an unspecified function. Traditional quantile single-index models, like classical quantile regression, struggle with inflated zeros, requiring further refinement. Our approach explicitly accounts for zero inflation, offering greater flexibility and precision in modeling response-covariate relationships. We adopt a semi-parametric framework instead of traditional non-parametric quantile regression to maintain model generality—allowing $G$ to remain unspecified—while enabling hypothesis testing for a subset of covariates of interest. In contrast, non-parametric methods often lack the ability to test specific covariates while adjusting for others.

The paper is organized as follows: Section 2 introduces our model and test statistics. Section 3 presents simulations comparing ZIQ-SIR with existing zero-inflated models, demonstrating its high power and well-controlled type I errors. In Section 4, we apply ZIQ-SIR to the Columbian Gut dataset, identifying more taxa associated with biological and dietary features than ZIQRank. Finally, Section 5 concludes with a discussion of potential future directions.

## 2 METHODS

We denote $Y$ as a non-negative zero-inflated response, and ${\bf Z}$ as a set of $p$ covariates of interest. We assume a set of additional $q$ covariates that need to be adjusted in the model but not of interest, and denote it as ${\bf C}$. The intercept is contained in ${\bf C}$. Throughout the paper, we denote $Q_Y(\tau \mid {\bf X})$ as the $\tau$th conditional quantile of $Y$ given ${\bf X} = ({\bf Z}^\top ,{\bf C}^\top )^\top$.

### 2.1 Zero-inflated quantile single-index model

To interpret the distribution of $Y$, we first decompose the conditional distribution of the zero-inflated response $Y$ into the zero part and the positive part:


\begin{eqnarray*}
&& P(Y \le y \mid \mathbf {X})\\
&=& P(Y = 0 \mid \mathbf {X}) + P(Y \le y \mid \mathbf {X}, Y > 0) P(Y > 0 \mid \mathbf {X}),\\
&& \text{for } y \ge 0,
\end{eqnarray*}

where $P(Y = 0 \mid \mathbf {X})$ represents the probability mass at zero, and $P(Y \le y \mid \mathbf {X}, Y > 0)$ corresponds to the conditional distribution of $Y$ given that $Y$ is positive. Then we model the two parts $P(Y=0\mid {\bf X})$ and $P(Y \le y \mid \mathbf {X}, Y > 0)$ separately. For $P(Y>0\mid {\bf X})$, the conditional probability of observing a positive $Y$, follows a logistic regression model,


(1) \begin{eqnarray*}
{\rm logit}\lbrace P(Y>0\mid {\bf X})\rbrace = {\bf Z}^\top \boldsymbol {\eta } + {\bf C}^\top \boldsymbol {\gamma },
\end{eqnarray*}

where $\boldsymbol {\eta }\in \mathbb {R}^p$, $\boldsymbol {\gamma }\in \mathbb {R}^q$ are the effects of covariates on the presence-absence status of the response. Then, we model the $\tau$th conditional quantile function of $Y$ given ${\bf X}$ and $Y>0$ by a semiparametric single-index model:


(2) \begin{eqnarray*}
Q_Y(\tau \mid {\bf X}, Y>0) = G_{\tau }({\bf Z}^\top \boldsymbol \alpha _{\tau }^0+{\bf C}^\top \boldsymbol \beta _{\tau }^0),
\end{eqnarray*}

where $G_{\tau }(\cdot )$ is an unknown function, and $\boldsymbol \alpha _{\tau }^0$, $\boldsymbol \beta _{\tau }^0$ are unknown parameters. The coefficient $\boldsymbol \alpha _{\tau }^0$ describes the effects of the covariates of interest on the response and $\boldsymbol \beta _{\tau }^0$ captures the contribution of other covariates.

To test whether the covariates of interest ${\bf Z}$ are associated with $Y$, the null hypothesis can be written as $H_0: \boldsymbol \eta = {\bf 0}\quad \text{and}\quad \boldsymbol \alpha _\tau ^0 = {\bf 0},\ \forall \tau \in (0,1).$ The first part, $\boldsymbol \eta = {\bf 0}$, can be tested through the likelihood ratio test (Nam et al., 2017). Thus, our main goal is to construct the test statistic for the second part, $\boldsymbol \alpha _\tau ^0$. In the quantile regression literature, the rank score test has been widely considered as a robust and powerful test for the quantile coefficient $\boldsymbol \alpha _\tau ^0$ (Gutenbrunner et al., 1993). To construct a rank score test statistic, we first introduce how to estimate the $G_\tau (\cdot )$ function. We approximate $G_{\tau }\left({\bf Z}^\top \boldsymbol \alpha _{\tau }^0+{\bf C}^\top \boldsymbol \beta _{\tau }^0\right)$ by B-spline as follows:


(3) \begin{eqnarray*}
G_{\tau }({\bf Z}^\top \boldsymbol \alpha _{\tau }^0+{\bf C}^\top \boldsymbol \beta _{\tau }^0)\approx B^\top ({\bf Z}^\top \boldsymbol \alpha _{\tau }+{\bf C}^\top \boldsymbol \beta _{\tau })\boldsymbol \theta (\tau ),
\end{eqnarray*}

where $\boldsymbol \theta (\tau )\in \mathbb {R}^{J_n}$ is an unknown parameter, $\boldsymbol \alpha _{\tau }$, $\boldsymbol \beta _{\tau }$ are unknown coefficients, and $B(u) = \lbrace B_j(u):1\le j\le J_n,J_n=N_{n}+m\rbrace ^\top$, a normalized $m$th order B-spline basis with a partition of $a=t_0 < t_1<...< t_{N_n}< b=t_{N_n+1}$ on a given interval $[a,b]$ (De Boor, 2001).

Suppose we have independent and identically distributed random samples $\lbrace \left({\bf X}_i,Y_i\right); i = 1,2,\cdots ,n\rbrace =\lbrace (({\bf Z}_i^\top ,{\bf C}_i^\top )^\top ,Y_i); i = 1,2,...,n\rbrace$. By models (2) and (3), we estimate the quantile coefficients by minimizing the following pseudo-likelihood function:


(4) \begin{eqnarray*}
&& L_{\tau n}(\boldsymbol \alpha _\tau ,\boldsymbol \beta _\tau ,\boldsymbol \theta _\tau )\\
&=&\frac{1}{n}\sum _{i=1}^n\rho _{\tau }\left\lbrace Y_i-B\left({\bf Z}_i^\top \boldsymbol \alpha _\tau +{\bf C}_i^\top \boldsymbol \beta _\tau \right)^\top \boldsymbol \theta _\tau \right\rbrace \\
&& I(Y_i>0),
\end{eqnarray*}

where $\rho _\tau (u) = u \lbrace \tau - I(u < 0)\rbrace $ represents the quantile loss function.

### 2.2 Estimation

The parameters $\boldsymbol \theta _\tau ,\boldsymbol \alpha _\tau ,\boldsymbol \beta _\tau$ do not have explicit forms of solutions and require numerical iteration methods for solving. We use the profile approach to iteratively estimate $\boldsymbol \theta _\tau$, $\boldsymbol \alpha _\tau$ and $\boldsymbol \beta _\tau$ due to its stable estimation performance (Liang et al., 2010; Ma and He, 2016). We define the profile pseudo-likelihood function of $\boldsymbol \alpha _\tau$ and $\boldsymbol \beta _\tau$ as


(5) \begin{eqnarray*}
L_{\tau n}^{*}(\boldsymbol \alpha _\tau , \boldsymbol \beta _\tau )&=&\min _{\boldsymbol \theta _\tau \in \mathbb {R}^{J_n}} L_{\tau n}(\boldsymbol \alpha _\tau , \boldsymbol \beta _\tau ,\boldsymbol \theta _\tau )=L_{\tau n}\left(\boldsymbol \alpha _\tau , \boldsymbol \beta _\tau ,\boldsymbol {\tilde{\theta }}_n\left(\boldsymbol \alpha _\tau , \boldsymbol \beta _\tau ,\tau \right)\right) \\
&=&\frac{1}{n}\sum _{i=1}^n\rho _{\tau }\left\lbrace Y_i-B\left({\bf Z}_i^\top \boldsymbol \alpha _\tau +{\bf C}_i^\top \boldsymbol \beta _\tau \right)^\top \boldsymbol {\tilde{\theta }}_n\left(\boldsymbol \alpha _\tau ,\boldsymbol \beta _\tau ,\tau \right)\right\rbrace\\
&&\times I(Y_i>0),
\end{eqnarray*}

where $\boldsymbol {\tilde{\theta }}_n\left(\boldsymbol \alpha _\tau , \boldsymbol \beta _\tau ,\tau \right)$ is the minimizer of $L_{\tau n}(\boldsymbol \alpha _\tau , \boldsymbol \beta _\tau ,\boldsymbol \theta _\tau )$ over $\boldsymbol \theta _\tau \in \mathbb {R}^{J_n}$ for given $\boldsymbol \alpha _\tau , \boldsymbol \beta _\tau$. Note that one cannot construct the rank score of the coefficient $\boldsymbol \alpha _\tau$ by differentiating the quantile objective function $L_{\tau n}^{*}(\boldsymbol \alpha _\tau ,\boldsymbol \beta _\tau )$ directly due to the lack of differentiability of $\tilde{\boldsymbol \theta }_n({\boldsymbol \alpha }_\tau , {\boldsymbol \beta }_\tau , \tau )$ as a function of ${\boldsymbol \alpha }_\tau$. We consider a smooth version of the profile quantile loss function:


(6) \begin{eqnarray*}
&&\tilde{L}_{\tau n}^{*}(\boldsymbol \alpha _\tau ,\boldsymbol \beta _\tau )\\
&=& \frac{1}{n}\sum _{i=1}^{n}\rho _{\tau }\left\lbrace Y_i-B\left({\bf Z}_i^\top \boldsymbol \alpha _\tau +{\bf C}_i^\top \boldsymbol \beta _\tau \right)^\top \tilde{\tilde{\boldsymbol \theta }}_n\left(\boldsymbol \alpha _\tau , \boldsymbol \beta _\tau ,\tau \right)\right\rbrace \\
&& I(Y_i>0),
\end{eqnarray*}

where the smoothness of ${\tilde{\tilde{\boldsymbol \theta }}}_{n}(\boldsymbol \alpha _\tau , \boldsymbol \beta _\tau ,\tau ) = \arg \min _{\boldsymbol \theta _\tau \in \mathbb {R}^{J_n}}\mathbb {E}\lbrace L_{\tau n}(\boldsymbol \theta _\tau ,\boldsymbol \alpha _\tau ,\boldsymbol \beta _\tau ) \mid \mathbb {X}\rbrace$ can be derived from Assumption (3.4), Supplement A.1.1, and $\mathbb {X} = ({\bf X}_1,\cdots ,{\bf X}_n)$ are the given covariates. With the profile likelihood function defined in eq (5), we can obtain the estimation of $ \left({\boldsymbol {\alpha }^0_\tau }^\top , {\boldsymbol \beta ^0_\tau }^\top \right)^\top$ under the null hypothesis: $\widehat{\boldsymbol \alpha }^{N}_\tau = {\bf 0}_{p\times 1},\quad {\widehat{\boldsymbol \beta }^{N}_\tau }= \arg \min _{\Vert \boldsymbol \beta _\tau \Vert _2 = 1}L_{\tau n}^{*}({\bf 0}_{p\times 1},\boldsymbol \beta _\tau ),$ and the spline estimator of the $G_\tau (\cdot )$ function is: $\widehat{G}_{\tau }\left(u\right) = B(u)^\top \boldsymbol {\tilde{\theta }}_n\left(\widehat{\boldsymbol \alpha }^N_\tau , \widehat{\boldsymbol \beta }^N_\tau ,\tau \right)$, where $\boldsymbol {\tilde{\theta }}_n\left(\widehat{\boldsymbol \alpha }^N_\tau , \widehat{\boldsymbol \beta }^N_\tau ,\tau \right)$ is the minimizer of $L_{\tau n}(\widehat{\boldsymbol \alpha }^N_\tau , \widehat{\boldsymbol \beta }^N_\tau ,\boldsymbol \theta _\tau )$ over $\boldsymbol \theta _\tau \in \mathbb {R}^{J_n}$.

### 2.3 Proposed test statistics and asymptotic properties

Since we have obtained the profile likelihood function and the estimator of $G_\tau (\cdot )$, we can construct the rank score test for quantile regression coefficient(s) $\boldsymbol \alpha _\tau ^0$ by differentiating the smooth quantile loss function $\tilde{L}_{\tau n}^{*}\left(\boldsymbol \alpha _\tau ,\boldsymbol \beta _\tau \right)$ in eq (6): ${\bf s}\left(\widehat{\boldsymbol {\alpha }}_\tau ^N,\widehat{\boldsymbol {\beta }}_\tau ^N\right)_{p\times 1}= -\partial \tilde{L}^{*}_{\tau n} \left(\widehat{\boldsymbol {\alpha }}_\tau ^N,\widehat{\boldsymbol {\beta }}_\tau ^N\right)/\partial \boldsymbol \alpha _\tau .$ Though the exact value of ${\bf s}\left(\boldsymbol {\widehat{\alpha }}_\tau ^N,\boldsymbol {\widehat{\beta }}_\tau ^N\right)$ cannot be directly obtained, since $\tilde{L}^{*}_{\tau n}$ involves the expectation of the pseudo-likelihood function, we can construct the empirical score to approximate: $\widehat{{\bf s}}\left(\widehat{\boldsymbol {\alpha }}_\tau ^N,\widehat{\boldsymbol {\beta }}_\tau ^N\right)= n^{-1}\sum _{i = 1}^{n}\rho _{\tau }^{(1)}\left\lbrace Y_{i}-\widehat{G}_\tau \left({\bf C}_{i}^\top \widehat{\boldsymbol \beta }_\tau ^N\right)\right\rbrace \left\lbrace \widehat{G}_{\tau }^{(1)}\left({\bf C}_{i}^\top \widehat{\boldsymbol \beta }_\tau ^N\right)\widehat{{\bf Z}}_i\left(\widehat{\boldsymbol \beta }_\tau ^N\right)\right.\\ \left. I(Y_i>0)\right\rbrace ,$ where $\rho _{\tau }^{(1)}(u) =\tau -I(u<0)$ denotes the first order derivative of the quantile loss function; $\widehat{G}_{\tau }^{(1)}\left({\bf C}_{i}^\top \widehat{\boldsymbol \beta }_\tau ^N\right) = B^{(1)}\left({\bf C}_{i}^\top \widehat{\boldsymbol \beta }_\tau ^N\right)^\top \tilde{\boldsymbol \theta }_n\left({\bf 0}_{p\times 1},\widehat{\boldsymbol \beta }_\tau ^N,\tau \right)$ denotes the spline estimator of the first-order derivative of $G_\tau \left(\cdot \right)$ under null hypothesis; and $(\widehat{{\bf Z}}_{i}(\boldsymbol \beta _\tau )^\top ,\widehat{{\bf C}}_{i}(\boldsymbol \beta _\tau )^\top )^\top = \widehat{{\bf X}}_{i}(\boldsymbol \beta _\tau ) = {\bf X}_{i} - \widehat{E}\left({\bf X}_{i}\mid {\bf C}_{i}^\top \boldsymbol \beta _\tau \right),$ where $\widehat{E}\left({\bf X}_{i}\mid {\bf C}_{i}^\top \widehat{\boldsymbol \beta }_{\tau }^N\right)$ is the estimator of $E\left({\bf X}_i\mid {\bf C}_i^\top \boldsymbol \beta _\tau ^0 \right)$ with


\begin{eqnarray*}
\widehat{E}\left({\bf X}_{i}\mid {\bf C}_{i}^\top \widehat{\boldsymbol \beta }_{\tau }^N\right) &=&B({\bf C}_{i}^\top \widehat{\boldsymbol \beta }_{\tau }^N)^\top \left\lbrace \sum _{i = 1}^{n} B\left({\bf C}_{i}^\top \widehat{\boldsymbol \beta }_{\tau }^N\right)B\left({\bf C}_{i}^\top \widehat{\boldsymbol \beta }_{\tau }^N\right)^\top I(Y_i>0) \right\rbrace ^{-1}\\
&&\quad \times \left\lbrace \sum _{i = 1}^n B\left({\bf C}_{i}^\top \widehat{\boldsymbol \beta }_{\tau }^N\right){\bf X}_{i}I(Y_i>0)\right\rbrace .
\end{eqnarray*}

We further denote $\widehat{\boldsymbol \Omega }_{\tau }$ to approximate the variance-covariance matrix for the empirical score constructed above: $$\widehat{\boldsymbol \Omega }_{\tau } = n^{-1}\sum _{i = 1}^{n}\widehat{{\bf g}}_{\tau ,i}\widehat{ {\bf g}}_{\tau , i}^\top = \begin{pmatrix}\widehat{\boldsymbol \Omega }_{\tau 11} \quad & \widehat{\boldsymbol \Omega }_{\tau 12} \\\widehat{\boldsymbol \Omega }_{\tau 21}\quad & \widehat{\boldsymbol \Omega }_{\tau 22} \end{pmatrix}$$, where $\widehat{ {\bf g}}_{\tau ,i} = \widehat{G}_{\tau }^{(1)}\left({\bf C}_{i}^\top \widehat{\boldsymbol \beta }_\tau ^N\right)\widehat{{\bf X}}_{i}(\widehat{\boldsymbol \beta }_\tau ^N)I(Y_i>0) = (\widehat{ {\bf g}}_{\tau , i 1}^\top ,\widehat{ {\bf g}}_{\tau , i2}^\top )^\top ,$ and $\widehat{\boldsymbol \Omega }_{\tau ll^{^{\prime }}} = n^{-1}\sum _{i = 1}^{n}\widehat{ {\bf g}}_{\tau , il}\widehat{ {\bf g}}_{\tau , il^{^{\prime }}}^\top$ for $l,l^{^{\prime }} = 1,2.$ With the empirical score $\widehat{{\bf s}}\left(\widehat{\boldsymbol {\alpha }}_\tau ^N,\widehat{\boldsymbol {\beta }}_\tau ^N\right)$ and the estimated covariance matrix $\widehat{\boldsymbol \Omega }_{\tau }$, the test statistic for the null hypothesis $\boldsymbol \alpha ^0_\tau = {\bf 0}_{p\times 1}$ can be constructed as


\begin{eqnarray*}
\mathcal {T}_{\tau } &=& n\left\lbrace \tau (1-\tau )\right\rbrace ^{-1}\left\lbrace \widehat{{\bf s}}\left(\boldsymbol {\widehat{\alpha }}_\tau ^N,\boldsymbol {\widehat{\beta }}_\tau ^N\right)\right\rbrace ^\top \left(\widehat{\boldsymbol \Omega }_{\tau 22} - \widehat{\boldsymbol \Omega }_{\tau 21}^\top \widehat{\boldsymbol \Omega }_{\tau 11}^+\widehat{\boldsymbol \Omega }_{\tau 12}\right)^+\left\lbrace \widehat{{\bf s}}\left(\boldsymbol {\widehat{\alpha }}_\tau ^N,\boldsymbol {\widehat{\beta }}_\tau ^N\right)\right\rbrace ,
\end{eqnarray*}

where $\left(\widehat{\boldsymbol \Omega }_{\tau 22} - \widehat{\boldsymbol \Omega }_{\tau 21}^\top \widehat{\boldsymbol \Omega }_{\tau 11}^+\widehat{\boldsymbol \Omega }_{\tau 12}\right)^+$ is an approximation of the inverse of the variance-covariance matrix of the empirical score $\widehat{{\bf s}}\left(\boldsymbol {\widehat{\alpha }}_\tau ^N,\boldsymbol {\widehat{\beta }}_\tau ^N\right)$. Note that $E\left[\lbrace \widehat{{\bf X}}_{i}(\widehat{\boldsymbol \beta }_\tau ^N)I(Y_i>0)\rbrace \lbrace \widehat{{\bf X}}_{i}(\widehat{\boldsymbol \beta }_\tau ^N)I(Y_i>0)\rbrace ^\top \right] = E\left[\widehat{{\bf X}}_{i}(\widehat{\boldsymbol \beta }_\tau ^N)\lbrace \widehat{{\bf X}}_{i}(\widehat{\boldsymbol \beta }_\tau ^N)\rbrace ^\top P(Y_i>0\mid {\bf X}_i)\right].$ The variance-covariance matrix $\left(\widehat{\boldsymbol \Omega }_{\tau 22} -\widehat{\boldsymbol \Omega }_{\tau 21}^\top \widehat{\boldsymbol \Omega }_{\tau 11}^+\widehat{\boldsymbol \Omega }_{\tau 12}\right)$ implicitly incorporates a “propensity score”, $P(Y_i>0\mid {\bf X}_i)$, which captures the uncertainty associated with whether $Y$ is observed as a positive count or remains zero. This reflects the randomness in the occurrence of positive counts in the data. With $n\rightarrow \infty$, we can derive the asymptotic distribution of the test statistic $\mathcal {T}_\tau$, which is proven in Supplement A.1.2.

Theorem 1: Under the null hypothesis, with Assumptions 1-3 in Supplement A.1.1 hold, given that $\left(Y,{\bf C}^\top \right)^\top$ is independent of ${\bf Z}$ given ${\bf C}^\top \boldsymbol \beta _\tau ^0$, as $n\rightarrow \infty$, we have $\mathcal {T}_{\tau }\stackrel{d}{\longrightarrow } \chi _{p}^2.$

Combining the tests of $\boldsymbol \eta$ and $\boldsymbol \alpha _\tau ^0$, we conclude a unified test for the following hypothesis: $H_0: \boldsymbol \eta = {\bf 0}\quad \text{and}\quad \boldsymbol \alpha ^0_\tau = {\bf 0},\ \forall \tau \in (0,1).$ We denote the $p$-value for logistic regression as $p^L$. For the quantile regression part, we conduct hypothesis testing for the coefficient $\boldsymbol \alpha _\tau ^0$ on a grid of $K$ quantile levels, $0<\tau _1<\cdots <\tau _K<1$, and obtain the $p$-values $p_{\tau _1}^Q, \cdots ,p_{\tau _K}^Q$. For computational efficiency, a common practice in quantile regression is to test $K=5$ levels $\tau \in \lbrace 0.1,0.25,0.5,0.75, 0.9\rbrace$. Finally, we combine $p^L$ and $p_{\tau _1}^Q, \cdots ,p_{\tau _K}^Q$ by Cauchy combination (Liu and Xie, 2020), and the final test statistic $\mathcal {T}_C$ takes the form $\mathcal {T}_C = \widehat{r}_n \tan \left\lbrace (0.5-p^L)\pi \right\rbrace + (1-\widehat{r}_n)\sum _{s = 1}^K w_s \tan \left\lbrace (0.5-p^Q_{\tau _s})\pi \right\rbrace ,$ where $\widehat{r}_n$ is the observed proportion of zero in $Y_i$’s and the weight $w_s = \frac{\tau _sI(\tau _s\le 0.5)+(1-\tau _s)I(\tau _s>0.5)}{\sum _{s = 1}^K\tau _sI(\tau _s\le 0.5)+(1-\tau _s)I(\tau _s>0.5)}$ are set to ensure that $p$-values with central quantiles are assigned with larger weight while the extreme tails are assigned with smaller weight (Ling et al., 2021a). The Cauchy combination is a flexible approach that does not require strong assumptions in practice. As established in Liu and Xie (2020) (Theorem 1 and Remark 1), it follows that $\mathcal {T}_C \stackrel{d}{\longrightarrow } {\it Cauchy}(0,1)$. Note that there are various options to combine multiple $p$-values other than the Cauchy combination, such as the minimum $p$-value method (Tippett et al., 1931), truncated product method (Zaykin et al., 2002), uniform combinations (Edgington, 1972). One can use any appropriate method, and we use the Cauchy combination here due to its fast computation.

The implementation details are provided in Supplement A.2, including a fast permutation strategy for small sample sizes and the knot selection for the B-spline component of ZIQ-SIR.

## 3 SIMULATIONS

Since the microbiome data often has a higher portion of zeros and smaller sample sizes than the dietary data, as reported in Section 1, modeling and testing become more challenging. Therefore, in this section, we simulate the dataset to mimic real microbiome data in Section 4. We compare ZIQ-SIR to four other methods: ZIQRank (Ling et al., 2021a), Quantile Single-index (Ma and He, 2016), ZINB (Ridout et al. (2001)), and ZIP (Yau et al. (2003)). For ZINB and ZIP, we first round the generated response to count data and use the Wald test in pscl package in R. The ZIQRank method can be viewed as a special case of ZIQ-SIR by specifying the function $G_{\tau }(\cdot )$ as an identity link function. The Quantile Single-index method performs similarly to the positive part of ZIQ-SIR but does not model the inflated zeros. When the data includes a probability mass at zero, the Quantile Single-index method fails to converge. To address this, we added a small perturbation $\left(N(0,10^{-10})\right)$ to the zero-valued responses before applying the Quantile Single-index method. We evaluate all methods based on their type I errors and power performance.

### 3.1 Simulation settings

We generate $Y$ to mimic microbiome abundance and ${\bf X}= (x_{1},x_{2},x_{3},x_{4},x_{5})^\top$ as covariates, based on real data distribution. For the covariates $x_{1},x_{2},x_{3},x_{4},x_{5}$, we generate ${\rm Setting}\ 1:\ x_{1}\sim Bernoulli(0.5), x_{2}\sim N(28,2^2), x_{3}\sim N(92.5,13^2), x_{4}\sim N(80,12^2), x_{5}\sim N(124,18.5^2);$ and ${\rm Setting}\ 2:\ x_{1}\sim Bernoulli(0.5),x_{2}\sim N(28,2^2),x_{3}\sim 2x_2+N(36.5,9^2), x_{4}\sim N(80,12^2)$, $x_{5}\sim 1.3x_4+N(20,7.75^2).$ We generate these covariates to mimic the real data in Section 4: $x_1$ represents gender, $x_2$ BMI, $x_3$ waist circumference, $x_4$ diastolic blood pressure, and $x_5$ systolic blood pressure. In Setting 1, all covariates are generated independently. In Setting 2, the correlations between $x_2$ and $x_3$, as well as $x_4$ and $x_5$, are simulated to mimic their real data correlations (see Table S.1 in Supplement B.1). For each subject ${\bf X}_i$, we first simulated its nominal quantile level $\tau _i\sim \text{Unif}(0,1)$ given the presence of the taxon, while $D_i$ is the presence-absence status from a Bernoulli distribution with a success probability defined as: $P(D = 1\mid {\bf X}) = \frac{\exp (\gamma _0+\sum _{j=1}^{p+q}\gamma _{j}x_{j})}{1+\exp (\gamma _0+\sum _{j=1}^{p+q}\gamma _{j}x_{j})},$ where $p$ denotes the dimension of covariates of interest, $q$ denotes the dimension of additional covariates, and the set of parameters $\boldsymbol \gamma = (-0.4,-0.480,-0.022,0.021,0.015, -0.009)^\top$ to mimic the taxon *Blautia* from real data analysis. We then set $Y_i= 0$ if $D_i=0$. Otherwise, we generated $Y_i$ as $Y_i = Q_Y(\tau _i\mid {\bf X}_i,Y>0) = G_{\tau _i}\left(\beta _0(\tau _i)+{\bf X}_i^\top \boldsymbol \beta (\tau _i)\right),$ where the true coefficients $\boldsymbol \beta (\tau ) = \left(\beta _1(\tau ),\cdots ,\beta _{p+q}(\tau )\right)^\top$ and the quantile function $G_{\tau }(\cdot )$ are generated as follows: $ \beta _0(\tau ) = -147.7\tau - 50\tau ^2-20, \beta _{1}(\tau ) = 0.6\sqrt{\tau }-2\tau , \beta _{2}(\tau ) = 2.2\tau ^2,\beta _3(\tau ) = \frac{1}{30}\tau ^2+0.02,$$\beta _{4}(\tau ) = 0.097\sin (2\pi \tau ), \beta _5(\tau ) = 0.086(-3\tau ^2+\tau ), G_{\tau }(u) = \frac{1}{12}\tau (0.1u)^4+13.5\tau (0.1u)^2 + 1.875\tau u.$ Setting 2 mimics the distribution of taxon *Blautia* as in the application from Section 4 (Figure S.1, Supplement B.1). Simulation results are presented based on 500 Monte Carlo replicates.

### 3.2 Type I error results

We first present the type I error results with sample sizes of $n=500,2000$. The samples $({\bf X}_i,Y_i)$, $i=1,\cdots ,n$ are generated under Setting 1 and Setting 2, respectively. We evaluate the type I error under the null model with the coefficients of $x_j$ set to zero, ie, $\beta _j(\tau ) = 0$ and $\gamma _j = 0$, for $j = 1,\cdots , p$, respectively. Additionally, in Setting 2, we further test whether the coefficients for the entire groups of correlated covariates $(x_2, x_3)$ and $(x_4, x_5)$ are zero.

The sample size of $n=500$ matches the real data size in Section 4 and can be viewed as a small sample case due to the inflated zeros in the data. On average, 36% of $Y_i$ generated under Setting 1 and 2 are zero. We apply the proposed ZIQ-SIR method using the fast permutation approach described in Supplement A.2. To ensure a fair and direct comparison with existing methods, we opted to use publicly available versions of ZIQRank and other competing methods without modification, rather than introducing permutation adjustments. From Table 1, we observe that our proposed ZIQ-SIR method maintains a controlled type I error, regardless of the correlations between covariates, while other methods all exhibit inflation to some extent. The ZINB and ZIP methods exhibit severe type I error inflation under both settings, as these parametric models fail to adequately describe the relationship between the covariates and $Y$ due to the overdispersion nature of the data. This inadequacy potentially leads to erroneous conclusions about the significant association between covariates of interest and $Y$ at a given confidence level. The Quantile Single-index method shows much more severe inflation when the covariates are correlated, possibly due to the violation of independence assumptions in Ma and He (2016). We also conducted additional type I error evaluation with a smaller sample size ($n=200$). The proposed ZIQ-SIR still maintains the type I error; see Table S.3, Supplement B.2.

**TABLE 1:** Type I error result with the significant threshold $\alpha =0.05$.

$n=500$	Predictor	ZIQ-SIR	ZIQRank	Quantile Single-index	ZINB	ZIP
Setting 1	$x_1$	0.056	0.074	0.090	0.880	0.904
	$x_2$	0.042	0.066	0.078	0.822	0.820
	$x_3$	0.054	0.064	0.090	0.902	0.888
	$x_4$	0.052	0.052	0.092	0.888	0.904
	$x_5$	0.046	0.062	0.082	0.906	0.888
Setting 2	$x_2$	0.044	0.082	0.088	0.850	0.834
	$x_3$	0.040	0.072	0.096	0.890	0.880
	$x_4$	0.044	0.066	0.314	0.908	0.896
	$x_5$	0.034	0.072	$ 0.330$	0.870	0.878
	$x_2$ , $x_3$	$0.052$	0.064	0.094	$0.970$	$0.946$
	$x_4$ , $x_5$	0.042	0.058	0.068	0.978	0.996

*n* = 2000	Predictor	ZIQ-SIR	ZIQRank	Quantile Single-index	ZINB	ZIP
Setting 1	$x_1$	0.046	0.056	0.048	0.902	0.948
	$x_2$	0.046	0.062	0.062	0.856	0.986
	$x_3$	0.044	0.050	0.072	0.906	0.932
	$x_4$	0.050	0.054	0.042	0.866	0.954
	$x_5$	0.050	0.058	0.068	0.932	0.940
Setting 2	$x_2$	0.058	0.058	0.066	0.798	0.988
	$x_3$	0.046	0.058	0.110	0.896	0.916
	$x_4$	0.056	0.062	0.326	0.912	0.974
	$x_5$	0.056	0.040	0.248	0.876	0.984
	$x_2$ , $x_3$	0.056	0.066	0.076	0.965	0.998
	$x_4$ , $x_5$	0.056	0.054	0.062	0.894	0.918

When the sample size increases to $n=2000$, we use the asymptotic distribution of $\mathcal {T}_\tau$ to obtain its $p$-value. We observe that the type I error of ZIQ-SIR is under control (Table 1), confirming the validity of asymptotic distribution. Note that type I error inflation persists for Quantile Single-Index and ZIQRank across different sample sizes due to the limited flexibility of their models. The type I error of ZIQRank is improved due to the increased sample size, similar to Quantile Single-index under Setting 1. However, their type I error is still inflated under Setting 2 due to the correlation among covariates and their model misspecifications.

The hypothesis testing results with a significance level of $\alpha = 0.01$ are consistent with the above results, provided in Supplement B.2 (Tables S.2 and S.4). We have also conducted additional simulation studies using hurdle Poisson and hurdle negative binomial methods (Mullahy, 1986). Similar to ZIP and ZINB, the hurdle methods also exhibit type I error inflation due to their restrictive parametric assumptions (Supplement B.3).

### 3.3 Power results

Power results are also performed under Setting 1 and Setting 2 with sample sizes of $n\in \lbrace 500,2000\rbrace$. We do not present the power results of ZINB and ZIP methods, since Table 1 shows their severe type I error inflation. The power for ZIQ-SIR, ZIQRank, and Quantile Single-index are presented in Table 2.

**TABLE 2:** Power results (without ZIP and ZINB) with the significant threshold $\alpha =0.05$.

$n=500$	Predictor	ZIQ-SIR	ZIQRank	Quantile Single-index
	$x_1$	0.484	0.474	0.124
	$x_2$	0.080	0.082	0.080
Setting 1	$x_3$	0.594	0.608	0.120
	$x_4$	0.276	0.278	0.098
	$x_5$	0.274	0.260	0.112
Setting 2	$x_2$	0.066	0.068	0.090
	$x_3$	0.330	0.286	0.126
	$x_4$	0.066	0.068	0.366
	$x_5$	0.068	0.082	0.384
	$x_2$ , $x_3$	0.292	0.270	0.116
	$x_4$ , $x_5$	0.082	0.074	0.110

*n* = 2000	Predictor	ZIQ-SIR	ZIQRank	Quantile Single-index
	$x_1$	0.998	0.992	0.386
	$x_2$	0.122	0.156	0.078
Setting 1	$x_3$	1.000	0.998	0.384
	$x_4$	0.916	0.910	0.210
	$x_5$	0.854	0.824	0.298
Setting 2	$x_2$	0.154	0.162	0.084
	$x_3$	0.934	0.906	0.180
	$x_4$	0.266	0.262	0.430
	$x_5$	0.194	0.190	0.426
	$x_2$ , $x_3$	0.914	0.886	0.084
	$x_4$ , $x_5$	0.218	0.198	0.096

Under Setting 1, with sample sizes of $n\in \lbrace 500,2000\rbrace$, we observe that our method demonstrates comparable power to the ZIQRank method, while the Quantile Single-index method shows significantly lower power. This suggests that ZIQ-SIR and ZIQRank better address the issue of zero inflation in the simulated data, thus achieving higher power. Under Setting 2, we observe that the power of the ZIQ-SIR method is generally higher than the ZIQRank method, suggesting that the ZIQ-SIR method better handles the correlation among covariates and results in a higher power. The Quantile Single-index method shows the highest power for certain covariates due to its type I error inflation (Table 1).

To better reflect real microbial taxonomic count data, we conduct additional simulations with count responses and present the results in Supplement B.4. These results, which account for the discrete nature of the data, remain consistent with our original findings.

## 4 APPLICATION IN COLUMBIAN’S GUT DATA

We demonstrate the performance of our ZIQ-SIR method using data from the Colombian Gut study (De la Cuesta-Zuluaga et al., 2018; Gonzalez et al., 2018). We present hypothesis testing results for taxa associated with health-related biological and dietary features using both ZIQ-SIR and ZIQRank. The results for ZIP, ZINB, and the Quantile Single-Index method are not included due to their significantly inflated type I error rates.

### 4.1 Data description

The dataset consists of microbiome counts for 425 taxa at the genus level for 441 adults, alongside covariates related to diet (eg, fiber, various fats, protein), anthropometric measures [age, body mass index (BMI)], lipid profile [high-density lipoprotein (HDL), LDL], glucose metabolism (glucose, insulin), blood pressure (diastolic and systolic), city, and medication usage. Categorical variables such as sex, medication, and city were treated as dummy variables. We excluded 3 subjects due to missing values and 2 others with triglyceride levels exceeding 800 mg/dL, resulting in a final sample size of 436.

We analyzed 109 taxa with observed zero proportions below 0.8, as higher percentages of zeros can lead to unreliable results (Zhang and Yi, 2020). Consistent with standard practice in microbiome studies (Xia et al., 2018), we adjusted for library size (ie, the total count of the 109 taxa per person) by including it as a covariate in our model. Other data normalization methods, such as rarefaction (Willis, 2019), relative abundance (Gloor et al., 2016), and CSS (McKnight et al., 2019), can also be applied. Additionally, since the quantile-based models are designed for continuous outcomes, we applied jittering (uniformly distributed between 0 and 1) to the non-zero microbiome counts.

The 24 covariates were divided into three groups: biological features, dietary features, and other covariates. Other covariates include 4 covariates: age, sex, city, and medication usage. The biological feature group comprises 12 covariates, including adiponectin, BMI, cholesterol, diastolic blood pressure, systolic blood pressure, glucose, glycosylated hemoglobin, HDL, LDL, insulin, triglycerides, and waist circumference. These covariates are closely linked to overall health (Ma and Shieh, 2006; Stefan et al., 2003). The dietary feature group includes 8 covariates related to macronutrient consumption: fiber, percentage of animal protein, carbohydrates, monounsaturated fat, polyunsaturated fat, saturated fat, total fat, and protein. We performed hypothesis testing for the 109 taxa against the biological and dietary feature groups using the proposed ZIQ-SIR method with a fast permutation approach (Supplement A.2), comparing the results with those by the ZIQRank method.

### 4.2 Permutation test for type I error evaluation

**FIGURE 3:**
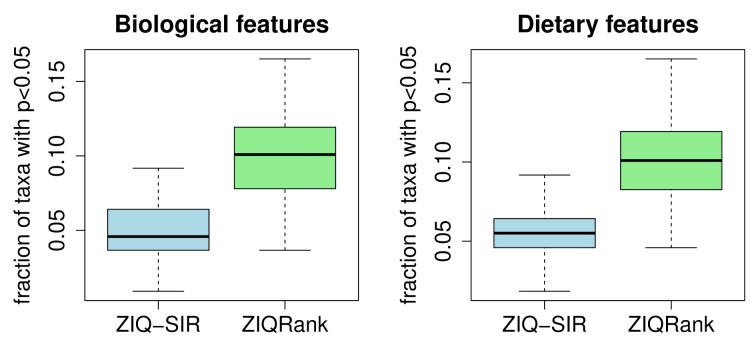
Boxplot of fraction of taxa with $p<0.05$ based on 50 null datasets.

As the effective sample size is relatively small due to excessive zeros, before analyzing the data using the two methods, we first evaluate the type I errors of ZIQ-SIR and ZIQRank on the real microbiome data testing for the biological features and dietary features. We permute the covariates jointly for each subject to create 50 null datasets. The permutation maintains the association between all the covariates but removes the association between covariates and the response, microbial abundance. Therefore, none of the covariates should have an impact on microbial abundance, and microbiomes with small $p$-values are considered false positives. We use the null datasets to test for the biological and dietary features, respectively, and report the type I error by the proportion of taxa with nominal $p$-values less than 0.05 within each set. This evaluation procedure is widely adopted in real data analysis (Ling et al., 2021b; Soneson and Robinson, 2018). Results suggest that, in both analyses, our method has type I error controlled with around 5% taxa having $p$-values smaller than 0.05, while the ZIQRank method has inflation to some extent (Figure 3).

### 4.3 Hypothesis testing results

We tested the relationship between microbial abundance and covariates of interest using the proposed ZIQ-SIR method and ZIQRank. The $p$-values are adjusted for multiple testing by controlling the False Discovery Rate (FDR) (Benjamini and Hochberg, 1995), and taxa with FDR-adjusted $p$-values less than 0.05 were considered significantly associated with the biological or dietary features. The detailed $p$-values are given in Table S.9, Supplement C.1.

Using the ZIQ-SIR method, we identified 3 taxa associated with the biological features at a 5% FDR threshold, including 1 taxon also identified by ZIQRank (Table S.9). *Peptococcaceae*-unspecified and *Rhizobiales*-unspecified-unspecified are exclusively discovered by our method. The literature suggests that *Peptococcaceae*-unspecified is closely related to LDL levels (Zhu et al., 2021) and lower triglyceride levels (Ejtahed et al., 2020), findings confirmed by our analysis. Similarly, *Rhizobiales*-unspecified-unspecified has been linked to glucose levels (Asensio et al., 2022). Both methods identified *Actinomycetales*-unspecified-unspecified, which has been reported to increase significantly in CAD patients with higher BMI, lower cholesterol, and higher glucose levels (Sawicka-Smiarowska et al., 2021).

For the dietary features, the ZIQ-SIR method uniquely identified the taxon *Peptostreptococcus* and found *Peptococcaceae*-unspecified in common with the ZIQRank method (Table S.9). Previous studies have indicated that low-fiber, high-protein diets influence the abundance of *Peptostreptococcus* (Martínez-López et al., 2021), while experimental evidence shows that *Peptococcaceae*-unspecified levels are significantly higher in individuals consuming high-fat diets (Wang et al., 2020). These findings are consistent with our analysis.

## 5 DISCUSSION

We proposed ZIQ-SIR to test associations between zero-inflated outcomes and covariates of interest, accommodating their potential nonlinear relationships. Our method introduces a new single-index model for zero-inflated data and provides detailed procedures for both large and small sample sizes. Numerical experiments show that ZIQ-SIR maintains well-controlled type I errors while achieving high power, whereas existing methods like ZINB, ZIP, and ZIQRank often exhibit inflated type I errors when their assumptions are violated.

While the single-index model in ZIQ-SIR provides greater flexibility for the positive component, the logistic regression component retains a linear structure, as real-world applications have not shown compelling evidence for a more complex formulation. Future work could explore more generalized logistic regression models (Stukel, 1988). Another promising future direction is adapting our method to test associations between covariates and groups of zero-inflated outcomes (eg, multiple taxa in microbiome studies), requiring methods for multivariate responses. This extension could reveal broader patterns and improve inference. Incorporating structural information, such as hierarchical or clustered relationships among outcomes, may further enhance model fit and statistical power (Washburne et al., 2018).

## Supplementary Material

ujaf050_Supplemental_Files Web Appendices, tables, figures, and code referenced in Sections  2, 3, and 4 are available with this paper at the Biometrics website on Oxford Academic.

## Data Availability

The dataset that supports the findings in this paper is available on https://qiita.ucsd.edu/ with Study ID 11993 (De la Cuesta-Zuluaga et al., 2018; Gonzalez et al., 2018).
