# Supplementary material for: A semiparametric quantile regression rank score test for zero-inflated data
Source: Biometrics. 2025 May 5;81(2):ujaf050. doi: 10.1093/biomtc/ujaf050 (PMC12050976; doi:10.1093/biomtc/ujaf050)
Supplement: ujaf050_Supplemental_Files — Web Appendices, tables, figures, and code referenced in Sections 2, 3, and 4 are available with this paper at the Biometrics website on Oxford Academic. [file ujaf050_supplemental_files.zip › ZIQSIR-sup.pdf]

BIOMETRICS 000, 000–000 DOI: 000
000 0000

Supplementary Materials for A Semiparametric Quantile Regression Rank Score Test for Zero-inflated Data by Zirui Wang, Wodan Ling and Tianying Wang

Zirui Wang*

Department of Statistics and Data Science, Tsinghua University, Beijing, 100084, China

**email:* wzr23@mails.tsinghua.edu.cn

and

Wodan Ling*

Division of Biostatistics, Department of Population Health Sciences, Weill Cornell Medicine, New York, 10065, USA

**email:* wol4002@med.cornell.edu

and

Tianying Wang*

Department of Statistics, Colorado State University, Fort Collins, 80523, USA

**email:* Tianying.Wang@colostate.edu

This paper has been submitted for consideration for publication in *Biometrics*

Supplement A: Technical details and implementation details for ZIQ-SIR

In this section, we present the technical details for our constructed statistics and the implementation details for our proposed method.

A.1 *Technical details for the asymptotic distribution of the test statistic $\mathcal{T}_\tau$*

A.1.1 *Assumptions.* We propose the assumptions below to guarantee the asymptotic distribution of the test statistic. Due to the nonparametric nature of G_τ, we first posit the following assumptions for model identifiability.

Assumption 1:

(1.1) The covariates $\mathbf{X} = (\mathbf{Z}^\top, \mathbf{C}^\top)^\top$ in our model satisfies that $\mathbf{X} \in \mathcal{C}$, where $\mathcal{C}$ is a compact set.

(1.2) $\boldsymbol{\beta}_\tau^0$ belongs to the parameter space $\boldsymbol{\Theta} = \left\{\boldsymbol{\beta} = (\beta_1, ..., \beta_q)^\top : \|\boldsymbol{\beta}\|_2 = 1, \beta_1 \geqslant 0, \boldsymbol{\beta} \in \mathbb{R}^q\right\}$ for identifiability. We further assume p and q are fixed and shall not increase with n.

(1.3) The support of the function G_τ is $\left[\inf\left(\mathbf{Z}^\top\boldsymbol{\alpha}_\tau^0 + \mathbf{C}^\top\boldsymbol{\beta}_\tau^0\right), \sup\left(\mathbf{Z}^\top\boldsymbol{\alpha}_\tau^0 + \mathbf{C}^\top\boldsymbol{\beta}_\tau^0\right)\right]$ for all $\mathbf{X} = \left(\mathbf{Z}^\top, \mathbf{C}^\top\right)^\top \in \mathcal{C}$, $\boldsymbol{\beta} \in \boldsymbol{\Theta}$ and $\boldsymbol{\alpha} = \mathbf{0}_{p\times 1}$.

The assumptions guarantee the identifiability of $\boldsymbol{\beta}_\tau^0$ for quantile single-index models (Ma and He, 2016). We then introduce some common assumptions on the distribution of zero-inflated data. We denote a_0 and b_0 to be the infimum and supremum of $\mathbf{Z}^\top\boldsymbol{\alpha}_\tau^0+\mathbf{C}^\top\boldsymbol{\beta}_\tau^0 = \mathbf{C}^\top\boldsymbol{\beta}_\tau^0$ over $\mathbf{X} \in \mathcal{C}$, where $\mathcal{C}$ is the compact set defined in Assumption 1 above.

Assumption 2:

(2.1) Observations $\{(\mathbf{X}_i, Y_i) = ((\mathbf{Z}_i^\top, \mathbf{C}_i^\top)^\top, Y_i); i = 1, 2, \cdots, n\}$ are i.i.d. from a joint distribution P, where $\mathbf{X}_i = (\mathbf{Z}_i^\top, \mathbf{C}_i^\top)^\top$, $\mathbf{Z}_i \in \mathbb{R}^p$ and $\mathbf{C}_i \in \mathbb{R}^q$.

(2.2) The conditional density of $f_Y(Y \mid \mathbf{X}, Y > 0)$ of Y given $X = \mathbf{X}$ and the condition that $Y > 0$ satisfies the Lipschitz condition of order 1 and we assume that $\sup_{\mathbf{X},Y} f_Y(Y \mid \mathbf{X}, Y > 0) < \infty$.

(2.3) The conditional quantile function has the property $\lim_{\tau\to 0^+} Q_Y(\tau \mid \mathbf{X}, Y>0) = 0$.

(2.4) $\forall \mathbf{X} \in \mathcal{C}$, we have $\|E(\mathbf{X}\mathbf{X}^\top)\|_\infty < \infty$.

Assumptions (2.1)-(2.2) are commonly used assumptions in the regression literature (Ling et al., 2022; Ma and He, 2016). Assumption (2.3) guarantees the continuity assumption of our proposed quantile function $Q(\tau \mid \mathbf{X})$. Assumptions (2.4) impose constraints on the quantities involved in the asymptotic distribution of the coefficients $\widehat{\boldsymbol{\beta}}_\tau^N$.

Since our proof concerns nonparametric smoothing literature, we first give some definitions and notations concerning this topic. Let $\mathcal{H}_r$ be the collection of all the functions on $[a_0, b_0]$ such that the mth order satisfies the Holder condition of order $r-m$, i.e. for any function $\phi \in \mathcal{H}_r$, there exists a constant C_0 s.t. $\left|\phi_\tau^{(m)}(u_1) - \phi_\tau^{(m)}(u_2)\right| \leqslant C_0|u_1-u_2|^{r-m}$, for any $u_1, u_2 \in [a_0, b_0]$. This collection of functions is essential for the proof of the convergence rate of the spline estimator of our function G_τ. Under the null hypothesis, we know that $\boldsymbol{\alpha}_\tau^0 = \mathbf{0}_{p\times 1}$. Therefore the function $G_\tau(\mathbf{Z}^\top\boldsymbol{\alpha}_\tau^0 + \mathbf{C}^\top\boldsymbol{\beta}_\tau^0)$ becomes $G_\tau(\mathbf{C}^\top\boldsymbol{\beta}_\tau^0)$. We present some assumptions on the coefficients $\boldsymbol{\alpha}_\tau^0, \boldsymbol{\beta}_\tau^0$ and the function $G_\tau(\cdot)$.

Assumption 3:

(3.1) The density function of $\mathbf{Z}^\top\boldsymbol{\alpha} + \mathbf{C}^\top\boldsymbol{\beta}$ is bounded away from zero and infinity on its support, for $(\boldsymbol{\alpha}^\top, \boldsymbol{\beta}^\top)^\top$ in a neighborhood of $(\mathbf{0}_{p\times 1}^\top, (\boldsymbol{\beta}_\tau^0)^\top)^\top$.

(3.2) The quantile coefficient function $\boldsymbol{\beta}_\tau^0$ is differentiable as a function of τ at $\forall \tau \in (0,1)$, and has bounded first derivative, i.e., $\sup_{\tau\in(0,1)} \dot{\boldsymbol{\beta}}_\tau^0 = \sup_{\tau\in(0,1)} \left.\dfrac{d\boldsymbol{\beta}_t^0}{dt}\right|_{t=\tau} < \infty$.

(3.3) There exists $r > \frac{3}{2}$, such that for any $\tau \in (0,1)$ we have $G_\tau \in \mathcal{H}_r$.

(3.4) We denote $\tilde{\tilde{G}}_{\tau n}(u, \boldsymbol{\alpha}, \boldsymbol{\beta}) = B(u)^\top \tilde{\tilde{\boldsymbol{\theta}}}_n(\boldsymbol{\alpha}, \boldsymbol{\beta}, \tau)$, where $\tilde{\tilde{\boldsymbol{\theta}}}_n(\boldsymbol{\alpha}, \boldsymbol{\beta}, \tau)$ is defined in eq (6) in Section 2.2. There exists a constant $c_0 \in (0, +\infty)$, such that

$$\sup_{\mathbb{X}} \left\|\partial\tilde{\tilde{G}}_{\tau n}(\mathbf{Z}_i^\top\boldsymbol{\alpha} + \mathbf{C}_i^\top\boldsymbol{\beta}, \boldsymbol{\alpha}, \boldsymbol{\beta})/\partial(\boldsymbol{\alpha}^\top, \boldsymbol{\beta}^\top)^\top - \partial\tilde{\tilde{G}}_{\tau n}(\mathbf{C}_i^\top\boldsymbol{\beta}_\tau^0, \mathbf{0}, \boldsymbol{\beta}_\tau^0)/\partial(\boldsymbol{\alpha}^\top, \boldsymbol{\beta}^\top)^\top\right\|_2$$
$$\leqslant c_0(\|\boldsymbol{\beta} - \boldsymbol{\beta}_\tau^0\|_2 + \|\boldsymbol{\alpha}\|_2).$$

for any $(\boldsymbol{\alpha},\boldsymbol{\beta})$ in the neighborhood of $(\boldsymbol{\alpha}_\tau^0,\boldsymbol{\beta}_\tau^0)$ and $\tau\in(0,1)$.

We also define $E^*(\mathbf{X}\mid \mathbf{C}^\top\boldsymbol{\beta}_\tau^0)=\frac{\mathbb{E}\{f_{\varepsilon_{\tau_S}}(0|\mathbf{X})\mathbf{X}|\mathbf{C}^\top\boldsymbol{\beta}_\tau^0\}}{\mathbb{E}\{f_{\varepsilon_\tau}(0|\mathbf{X})|\mathbf{C}^\top\boldsymbol{\beta}_\tau^0\}}$, and

$$\widetilde{\mathbf{X}}=\mathbf{X}-E^*(\mathbf{X}\mid \mathbf{C}^\top\boldsymbol{\beta}_\tau^0), \tag{S.1}$$

where $f_{\varepsilon_\tau}(\varepsilon\mid\mathbf{X})$, satisfying that $f_{\varepsilon_\tau}(0\mid\mathbf{X})=f_{\varepsilon_\tau}(0)$, denotes the conditional density of ε_τ given $\mathbf{x}$, and $\varepsilon_\tau=Y-G_\tau(\mathbf{C}^\top\boldsymbol{\beta}_\tau^0)$ given $Y>0$.

(3.5) For any $\tau\in(0,1)$, $E^*(\mathbf{X}\mid\mathbf{C}^\top\boldsymbol{\beta}_\tau^0=u)$, which is a function of u, has a continuous and bounded first derivative.

Assumption (3.1), (3.3) and (3.5) are commonly used in the nonparametric smoothing literature (Cui et al., 2011; He and Shi, 1996). Assumption (3.2) is necessary for deducing the asymptotic behavior of estimated coefficient $\widehat{\boldsymbol{\beta}}_\tau^N$, which is important for establishing the asymptotic distribution of the test statistic $\mathcal{T}_\tau$. Assumption (3.4) is a typical assumption in the regression literature, which can be easily satisfied when the dimension of covariates is fixed.

A.1.2 *Proof of Theorem 1.* For a specific τ, under the null hypothesis, we know that $\boldsymbol{\alpha}_\tau^0=\mathbf{0}_{p\times1}$. We denote $g_{\tau,i1}=G_\tau^{(1)}\left(\mathbf{C}_i^\top\boldsymbol{\beta}_\tau^0\right)\widetilde{\mathbf{C}}_i$, $g_{\tau,i2}=G_\tau^{(1)}\left(\mathbf{C}_i^\top\boldsymbol{\beta}_\tau^0\right)\widetilde{\mathbf{Z}}_i$, $g_{\tau,i}=G_\tau^{(1)}\left(\mathbf{C}_i^\top\boldsymbol{\beta}_\tau^0\right)\widetilde{\mathbf{X}}_i$, and

$$\boldsymbol{\Omega}=E\left(I(Y_i>0)\left(g_{\tau,i}g_{\tau,i}^\top\right)\right)=\begin{pmatrix}\boldsymbol{\Omega}_{11} & \boldsymbol{\Omega}_{12}\\ \boldsymbol{\Omega}_{21} & \boldsymbol{\Omega}_{22}\end{pmatrix},$$

where $\left(\widetilde{\mathbf{Z}}^\top,\widetilde{\mathbf{C}}^\top\right)^\top=\widetilde{\mathbf{X}}$ is define in equation (S.1). Let $\boldsymbol{\Omega}_{nll'}=n^{-1}\sum_{i=1}^n I(Y_i>0)\left(g_{\tau,il}g_{\tau,il'}^\top\right)$ for $l,l'=1,2$. First, we consider:

$$\widetilde{\mathbf{s}}^*\left(\boldsymbol{\beta}_\tau^0\right) \;=\; n^{-1}\sum_{i=1}^n\left(g_{\tau,i2}-\boldsymbol{\Omega}_{21}\boldsymbol{\Omega}_{11}^+g_{\tau,i1}\right)\times\rho_\tau^{(1)}\left\{Y_i-G_\tau\left(\mathbf{C}_i^\top\boldsymbol{\beta}_\tau^0\right)\right\}I(Y_i>0).$$

Let $f_{\tau,i}=\left(g_{\tau,i2}-\boldsymbol{\Omega}_{21}\boldsymbol{\Omega}_{11}^+g_{\tau,i1}\right)\rho_\tau^{(1)}\left\{Y_i-G_\tau\left(\mathbf{C}_i^\top\boldsymbol{\beta}_\tau^0\right)\right\}I(Y_i>0)$. Then we have: $\widetilde{\mathbf{s}}^*\left(\boldsymbol{\beta}_\tau^0\right)=n^{-1}\sum_{i=1}^n f_{\tau,i}$. Since $E\{f_{\tau,i}\}=E\{E\{f_{\tau,i}\mid I(Y_i>0)\}\}=0$, we have $E\left\{\sqrt{n}\widetilde{\mathbf{s}}^*\left(\boldsymbol{\beta}_\tau^0\right)\right\}=0$.

Similar to Ma and He (2016), by central limit theorem we have:

$$\sqrt{n}\widetilde{\mathbf{s}}^*\left(\boldsymbol{\beta}_\tau^0\right) \xrightarrow{P} N\left(\mathbf{0}_p, \tau(1-\tau)(\boldsymbol{\Omega}_{22}-\boldsymbol{\Omega}_{21}\boldsymbol{\Omega}_{11}^+\boldsymbol{\Omega}_{12})\right).$$

Replacing $\boldsymbol{\Omega}_{ll'}$ by $\boldsymbol{\Omega}_{nll'}$ for $l,l'=1,2$ in $\widetilde{\mathbf{s}}^*\left(\boldsymbol{\beta}_\tau^0\right)$, we then define

$$\widetilde{\mathbf{s}}\left(\boldsymbol{\beta}_\tau^0\right) = n^{-1}\sum_{i=1}^n\left(g_{\tau,i2}-\boldsymbol{\Omega}_{n21}\boldsymbol{\Omega}_{n11}^+g_{\tau,i1}\right)\times\rho_\tau^{(1)}\left\{Y_i-G_\tau\left(\mathbf{C}_i^\top\boldsymbol{\beta}_\tau^0\right)\right\}I(Y_i>0).$$

With the fact that $\boldsymbol{\Omega}_{n21}\boldsymbol{\Omega}_{n11}^+=\boldsymbol{\Omega}_{21}\boldsymbol{\Omega}_{11}^++o_p(1)$, we can conclude by calculation:

$$\left\|\widetilde{\mathbf{s}}\left(\boldsymbol{\beta}_\tau^0\right)-\widetilde{\mathbf{s}}^*\left(\boldsymbol{\beta}_\tau^0\right)\right\|_2=o_p(n^{-1/2}).$$

To deduce the asymptotic distribution of $\mathcal{T}_\tau$, we only need to show that:

$$\left\|\widetilde{\mathbf{s}}\left(\boldsymbol{\beta}_\tau^0\right)-\widehat{\mathbf{s}}\left(\widehat{\boldsymbol{\alpha}}_\tau^N,\widehat{\boldsymbol{\beta}}_\tau^N\right)\right\|_2=o_p(n^{-1/2}) \tag{S.2}$$

$$\widehat{\boldsymbol{\Omega}}_n=\boldsymbol{\Omega}^{22}+o_p(1), \tag{S.3}$$

where $\boldsymbol{\Omega}^{22}=\left(\boldsymbol{\Omega}_{22}-\boldsymbol{\Omega}_{21}\boldsymbol{\Omega}_{11}^+\boldsymbol{\Omega}_{12}\right)^+$ and $\widehat{\boldsymbol{\Omega}}_n=\left(\widehat{\boldsymbol{\Omega}}_{\tau22}-\widehat{\boldsymbol{\Omega}}_{\tau21}^\top\widehat{\boldsymbol{\Omega}}_{\tau11}^+\widehat{\boldsymbol{\Omega}}_{\tau12}\right)^+$. By Assumption 2 and 3, we can establish the asymptotic distribution of the coefficient $\widehat{\boldsymbol{\beta}}_\tau^N$. Then with the asymptotic normality of $\widehat{\boldsymbol{\beta}}_\tau^N$ and equation (A.14) in Ma and He (2016), we can conclude that $\left|\widehat{G}_\tau^{(1)}\left(\mathbf{C}_i^\top\widehat{\boldsymbol{\beta}}_\tau^N\right)\widehat{\mathbf{X}}_i-G_\tau^{(1)}\left(\mathbf{C}_i^\top\boldsymbol{\beta}_\tau^0\right)\widetilde{\mathbf{X}}_i\right|=o_p(1)$, so that the equation (S.3) follows. For equation (S.2), we only need to show that:

$$\begin{aligned}A_n\left(\widehat{\boldsymbol{\beta}}_\tau^N\right)=&n^{-1}\sum_{i=1}^n\left[\rho_\tau^{(1)}\left\{Y_i-\widetilde{G}_{\tau n}\left(\mathbf{C}_i^\top\widehat{\boldsymbol{\beta}}_\tau^N,\widehat{\boldsymbol{\beta}}_\tau^N\right)\right\}-\rho_\tau^{(1)}\left\{Y_i-G_\tau\left(\mathbf{C}_i^\top\boldsymbol{\beta}_\tau^0\right)\right\}\right]I(Y_i>0)g_{\tau,i2}\\=&-n^{-1}\sum_{i=1}^n\boldsymbol{\Omega}_{n21}\boldsymbol{\Omega}_{n11}^+g_{\tau,i1}I(Y_i>0)\times\rho_\tau^{(1)}\left\{Y_i-G_\tau\left(\mathbf{C}_i^\top\boldsymbol{\beta}_\tau^0\right)\right\}+o_p\left(n^{-1/2}\right),\end{aligned} \tag{S.4}$$

and

$$\begin{aligned}C_n\left(\widehat{\boldsymbol{\beta}}_\tau^N\right)=&n^{-1}\sum_{i=1}^n\rho_\tau^{(1)}\left\{Y_i-\widetilde{G}_{\tau n}\left(\mathbf{C}_i^\top\widehat{\boldsymbol{\beta}}_\tau^N,\widehat{\boldsymbol{\beta}}_\tau^N\right)\right\}I(Y_i>0)\left\{g_{\tau,i2}-\widetilde{G}_{\tau n}^{(1)}\left(\mathbf{C}_i^\top\widehat{\boldsymbol{\beta}}_\tau^N,\widehat{\boldsymbol{\beta}}_\tau^N\right)\widehat{\mathbf{Z}}_i\left(\widehat{\boldsymbol{\beta}}_\tau^N\right)\right\}\\=&o_p\left(n^{-1/2}\right).\end{aligned} \tag{S.5}$$

We first break $A_n\left(\widehat{\boldsymbol{\beta}}_\tau^N\right)$ into two parts as below:

$$A_n\left(\widehat{\boldsymbol{\beta}}_\tau^N\right)=A_{n1}\left(\widehat{\boldsymbol{\beta}}_\tau^N,\tilde{\boldsymbol{\theta}}_n(\widehat{\boldsymbol{\alpha}}_\tau^N,\widehat{\boldsymbol{\beta}}_\tau^N,\tau)\right)+A_{n2}\left(\widehat{\boldsymbol{\beta}}_\tau^N\right),$$

where

$$
\begin{aligned}
A_{n1}(\boldsymbol{\beta},\boldsymbol{\theta}) &= n^{-1}\sum_{i=1}^{n} A_{n1,i}(\boldsymbol{\beta},\boldsymbol{\theta}) \\
&= n^{-1}\sum_{i=1}^{n}\left[\rho_\tau^{(1)}\left\{Y_i - B\left(\mathbf{C}_i^\top\boldsymbol{\beta}\right)^\top\boldsymbol{\theta}\right\} - \rho_\tau^{(1)}\left\{Y_i - \widetilde{G}_\tau\left(\mathbf{C}_i^\top\boldsymbol{\beta},\boldsymbol{\beta}\right)\right\}\right] I(Y_i>0)g_{\tau,i2},
\end{aligned}
$$

$$
\begin{aligned}
A_{n2}(\boldsymbol{\beta}) &= n^{-1}\sum_{i=1}^{n} A_{n2,i}(\boldsymbol{\beta}) \\
&= n^{-1}\sum_{i=1}^{n}\left[\rho_\tau^{(1)}\left\{Y_i - \widetilde{G}_\tau\left(\mathbf{C}_i^\top\boldsymbol{\beta},\boldsymbol{\beta}\right)\right\} - \rho_\tau^{(1)}\left\{Y_i - \widetilde{G}_\tau\left(\mathbf{C}_i^\top\boldsymbol{\beta}_\tau^0,\boldsymbol{\beta}_\tau^0\right)\right\}\right] I(Y_i>0)g_{\tau,i2}.
\end{aligned}
$$

Since $(Y_i,\mathbf{C}_i^\top)^\top$ and $\mathbf{Z}_i$ are independent given $\left(\mathbf{C}_i^\top\boldsymbol{\beta}_\tau^0\right)$,

$$
\left[\rho_\tau^{(1)}\left\{Y_i - \widetilde{G}_\tau\left(\mathbf{C}_i^\top\boldsymbol{\beta},\boldsymbol{\beta}\right)\right\} - \rho_\tau^{(1)}\left\{Y_i - \widetilde{G}_\tau\left(\mathbf{C}_i^\top\boldsymbol{\beta},\boldsymbol{\beta}\right)\right\}\right] I(Y_i>0)
$$

which is a function of Y_i and $\mathbf{C}_i^\top\boldsymbol{\beta}$, is independent of $\mathbf{Z}_i$ given $\mathbf{C}_i^\top\boldsymbol{\beta}_\tau^0$. Moreover, we have $E\left(g_{\tau,i2}\mid \mathbf{C}_i^\top\boldsymbol{\beta}_\tau^0\right)=0$. Consequently, we can deduce that:

$$
E\left(E\left(A_{n1}(\boldsymbol{\beta},\boldsymbol{\theta})\mid \mathbf{C}_i^\top\boldsymbol{\beta}_\tau^0\right)\right)=0. \tag{S.6}
$$

By the procedure in page S.26–S.27 of Ma et al. (2015), we have:

$$
A_{n1}\left(\widehat{\boldsymbol{\beta}}_\tau^N,\tilde{\boldsymbol{\theta}}_n(\widehat{\boldsymbol{\alpha}}_\tau^N,\widehat{\boldsymbol{\beta}}_\tau^N,\tau)\right)=o_p(n^{-1/2}). \tag{S.7}
$$

As equation (S.6), we also have: $E\left(A_{n2,i}\mid \mathbf{C}_i^\top\boldsymbol{\beta}_\tau^0\right)=0$. Using similar approach for proving equation (S.7), we can deduce from the Bernstein's inequality (Bosq, 1987) that: for any $\boldsymbol{\beta}$, s.t. $\|\boldsymbol{\beta}-\boldsymbol{\beta}_\tau^0\|_2=O(n^{-1/2})$,

$$
\left\|A_{n2}\left(\boldsymbol{\beta}\right)-\sum_{i=1}^{n}E\left[A_{n2,i}\left(\boldsymbol{\beta}\right)\mid \mathbf{X}_i\right]\right\|_2=o_p(n^{-1/2}). \tag{S.8}
$$

For sufficiently small $|t|$, we have:

$$
\begin{aligned}
& E\left\{\left(\rho_\tau^{(1)}(\varepsilon+t)-\rho_\tau^{(1)}(\varepsilon)\right)\times I(Y>0)\mid \mathbf{X}\right\} \\
= \;& P(Y=0)E\left\{\left(\rho_\tau^{(1)}(\varepsilon+t)-\rho_\tau^{(1)}(\varepsilon)\right)\times 0\mid \mathbf{X},Y=0\right\} \\
& +P(Y>0)E\left\{\left(\rho_\tau^{(1)}(\varepsilon+t)-\rho_\tau^{(1)}(\varepsilon)\right)\mid \mathbf{X},Y>0\right\} \\
= \;& \pi(\boldsymbol{\gamma},\mathbf{X})f_\varepsilon(0)t+o(|t|),
\end{aligned}
$$

where $\pi(\boldsymbol{\gamma}, \mathbf{X})$ denotes the probability of observing a positive Y given $\mathbf{X}$ according to the logistic regression model (1). By the equation above, we have:

$$\begin{aligned}
& n^{-1}\sum_{i=1}^{n} E\left[A_{n2,i}\left(\widehat{\boldsymbol{\beta}}_\tau^N\right) \mid \mathbf{X}_i\right] \\
= \;& n^{-1}\sum_{i=1}^{n} \pi(\boldsymbol{\gamma}, \mathbf{X}_i) f_\varepsilon(0) \times \left\{\widetilde{G}_\tau\left(\mathbf{C}_i^\top \widehat{\boldsymbol{\beta}}_\tau^N, \widehat{\boldsymbol{\beta}}_\tau^N\right) - \widetilde{G}_\tau\left(\mathbf{C}_i^\top \boldsymbol{\beta}_\tau^0, \boldsymbol{\beta}_\tau^0\right)\right\} g_{\tau,i2} + o_p(n^{-1/2}) \\
= \;& n^{-1}\sum_{i=1}^{n} g_{\tau,i2} \times \pi(\boldsymbol{\gamma}, \mathbf{X}_i) f_\varepsilon(0) G_\tau^{(1)}\left(\mathbf{C}_i^\top \boldsymbol{\beta}_\tau^0\right) \times \mathbf{C}_i^\top\left(\widehat{\boldsymbol{\beta}}_\tau^N - \boldsymbol{\beta}_\tau^0\right) + o_p(n^{-1/2}).
\end{aligned}$$

The last equation is given by Taylor expansion and the $\sqrt{n}$-convergence rate of $\widehat{\boldsymbol{\beta}}_\tau^N$. By equation (A.23) in Ma and He (2016), we have:

$$\begin{aligned}
& n^{-1}\sum_{i=1}^{n} E\left[A_{n2,i}\left(\widehat{\boldsymbol{\beta}}_\tau^N\right) \mid \mathbf{X}_i\right] \\
= \;& -n^{-1}\sum_{i=1}^{n} g_{\tau,i2} \times \pi(\boldsymbol{\gamma}, \mathbf{X}_i) f_\varepsilon(0) G_\tau^{(1)}\left(\mathbf{C}_i^\top \boldsymbol{\beta}_\tau^0\right) \times \widetilde{\mathbf{C}}_i^\top\left(\widehat{\boldsymbol{\beta}}_\tau^N - \boldsymbol{\beta}_\tau^0\right) + o_p(n^{-1/2}) \\
= \;& -n^{-1}\sum_{i=1}^{n} \pi(\boldsymbol{\gamma}, \mathbf{X}_i) f_\varepsilon(0) \times g_{\tau,i2} g_{\tau,i1}^\top\left(\widehat{\boldsymbol{\beta}}_\tau^N - \boldsymbol{\beta}_\tau^0\right) + o_p(n^{-1/2}). \qquad \text{(S.9)}
\end{aligned}$$

By (A.21) in Ma and He (2016), we have:

$$\widehat{\boldsymbol{\beta}}_\tau^N - \boldsymbol{\beta}_\tau^0 = f_\varepsilon(0)^{-1} \boldsymbol{\Omega}_{n11}^+ n^{-1}\sum_{i=1}^{n} g_{\tau,i1}\rho_\tau^{(1)}\left\{Y_i - G_\tau\left(\mathbf{X}_i^\top \boldsymbol{\beta}_\tau^0\right)\right\} + o_p\left(n^{-1/2}\right). \qquad \text{(S.10)}$$

By equation (S.9) and (S.10), we have:

$$\begin{aligned}
& n^{-1}\sum_{i=1}^{n} E\left[A_{n2,i}\left(\widehat{\boldsymbol{\beta}}_\tau^N\right) \mid \mathbf{X}_i\right] \\
& = -n^{-1}\sum_{i=1}^{n} \pi(\boldsymbol{\gamma}, \mathbf{X}_i) g_{\tau,i2} g_{\tau,i1}^\top \boldsymbol{\Omega}_{n11}^+ \times \left(n^{-1}\sum_{i=1}^{n} g_{\tau,i1}\rho_\tau^{(1)}\left\{Y_i - G_\tau\left(\mathbf{X}_i^\top \boldsymbol{\beta}_\tau^0\right)\right\}\right) \qquad \text{(S.11)} \\
& = -n^{-1} \boldsymbol{\Omega}_{n21} \boldsymbol{\Omega}_{n11}^+ n^{-1}\sum_{i=1}^{n} g_{\tau,i1}\rho_\tau^{(1)}\left\{Y_i - G_\tau\left(\mathbf{X}_i^\top \boldsymbol{\beta}_\tau^0\right)\right\} + o_p(n^{-1/2}).
\end{aligned}$$

The last equation comes from the property of the Bernoulli distribution of the covariate $I(Y_i > 0)$ given $\mathbf{X}_i$.

Therefore, we can conclude result (S.4) from equation (S.8) and (S.11). The equation (S.5) can be derived through a similar procedure.

A.2 *Implementation details*

Since checking the conditional independence of $\left(Y, \mathbf{C}^{\top}\right)^{\top}$ and $\mathbf{Z}$ given $\left(\mathbf{C}^{\top}\boldsymbol{\beta}_{\tau}^{0}\right)$ is challenging, we first project the additional covariates $\mathbf{C}$ onto the variables of interest $\mathbf{Z}$ before performing hypothesis testing with the ZIQ-SIR method. In cases where the sample size is insufficient, the Chi-square approximation for the test statistic $\mathcal{T}_{\tau}$ may not be reliable, given the sparse nature of zero-inflated data (Wang et al., 2022). To address this, we employ a fast permutation approach based on the Pearson Type III distribution to approximate the null permutation distribution as suggested in Wang et al. (2022); Zhan and Wu (2018). Specifically, we first decentralize the outer product of rank score: $\widehat{\mathbf{s}}\left(\widehat{\boldsymbol{\alpha}}_{\tau}^{N}, \widehat{\boldsymbol{\beta}}_{\tau}^{N}\right)\widehat{\mathbf{s}}\left(\widehat{\boldsymbol{\alpha}}_{\tau}^{N}, \widehat{\boldsymbol{\beta}}_{\tau}^{N}\right)^{\top}$ and the approximated variance-covariance matrix $\left(\widehat{\boldsymbol{\Omega}}_{\tau 22} - \widehat{\boldsymbol{\Omega}}_{\tau 21}^{\top}\widehat{\boldsymbol{\Omega}}_{\tau 11}^{+}\widehat{\boldsymbol{\Omega}}_{\tau 12}\right)^{+}$. Then, we construct the test statistic as the trace of the product of the two decentralized matrices and obtain the p-value by approximating its null distribution by a Pearson Type III. The validity of this fast permutation method has been demonstrated in prior studies (Wang et al., 2022; Zhan and Wu, 2018) and further corroborated by our own simulation results. This approach is applied directly to the estimated rank scores and the variance-covariance matrix, ensuring computational efficiency and robust performance in our analysis.

For B-spline estimation, we use equally spaced knots for the order m B-spline, with $N_n = \lfloor a n^{1/(2m+1)} \rfloor + 1$, where $\lfloor \cdot \rfloor$ denotes the integer part of a number and $a > 0$ is a constant when estimating the coefficient $\widehat{\boldsymbol{\beta}}_{\tau}^{N}$. The choice of constant a has minimal impact on the estimation within a reasonable range (Ma and He, 2016). In our numerical studies, we set $a = 1$. After estimating the coefficients, we select N_n by identifying the first local minimum of the following BIC criterion: $\mathrm{BIC}(N_n) = \log\left(L_{\tau n}^{*}\left(\widehat{\boldsymbol{\alpha}}_{\tau}^{N}, \widehat{\boldsymbol{\beta}}_{\tau}^{N}\right)\right) + \frac{\log(n)}{2n}\left(N_n + m\right)$.

Supplement B: Additional Simulation Results

B.1 *Real data evidence supporting simulation design*

We present the correlations of some covariates in real data in Table S.1 to support the correlated covariates in Setting 2 (Section 3.1).

[Table 1 about here.]

In Figures S.1, we present the taxon simulated by Setting 2 in Section 3. We can observe that the distributions of the simulated taxon (right panel) mimic the actual taxon's distribution (left panel).

[Figure 1 about here.]

B.2 *Additional simulation results for hypothesis testing*

We first present the type I error results under Setting 1 and Setting 2 with sample sizes of $n \in \{500, 2000\}$ and the significance level of $\alpha = 0.01$ (Table S.2, Table S.4). We can conclude that the type I error of our proposed ZIQ-SIR method is under control at the significance level $\alpha = 0.01$, regardless of the covariates' settings and the sample sizes, aligning with the results in the paper; while other methods all have inflation to some extent. We also present the type I errors under the two settings with a small sample size of $n = 200$ with the significance threshold $\alpha = 0.01$ and 0.05 (Table S.3), demonstrating that our method maintains the type I error even when the sample size is small.

[Table 2 about here.]

[Table 3 about here.]

[Table 4 about here.]

We then present the power results for Settings 1 and 2 with the significant threshold $\alpha = 0.01$ and the sample size of $n = 2000$ in Table S.5. Results suggest that the power of

ZIQ-SIR is generally higher than other methods given a controlled type I error, consistent with the power results with $\alpha = 0.05$ in Table 2.

[Table 5 about here.]

B.3 *Additional simulation results for hurdle models*

In this section, we present the type I error results for hurdle poisson (denoted as "H-Poisson") and hurdle negative binomial (denoted as "H-NB") under Settings 1-2 in Table S.6. For H-Poisson and H-NB, we use the `pscl` (Zeileis et al., 2008) package to fit the model and use the likelihood test in `lmtest` in `R` for hypothesis testing. From Table S.6, we can observe that the hurdle Poisson method presents severe type I error inflation regardless of sample size while hurdle negative binomial also results in severe inflation when $n \in \{500, 2000\}$. The restrictive parametric assumption of both methods made them unable to correctly identify the relationship between X and response Y, resulting in type I error inflation.

[Table 6 about here.]

B.4 *Additional simulation results for count data*

Since microbiome abundance is measured as an integer, we round the Y_i values generated in Settings 1-2 to better reflect real-world data. Here, we present the type I error and power results for three quantile-based methods, ZIQ-SIR, ZIQRank, and Quantile Single Index, using sample sizes of 500 and 2000. To satisfy the continuity condition required by quantile regression, we applied jittering (uniformly distributed between 0 and 1) to the nonzero microbiome counts (Ling et al., 2021), following the approach in Section 4. As shown in Tables S.7 and S.8, ZIQ-SIR improves power compared to ZIQRank while maintaining proper type I error control. In contrast, both ZIQRank and the Quantile Single Index method exhibit some degree of type I error inflation. These results align with those obtained when Y_i is continuous, as presented in the main text.

[Table 7 about here.]

[Table 8 about here.]

Supplement C: Detailed hypothesis testing results for Application (I)

This section presents detailed background information and additional hypothesis testing results for Section 4.

In Table S.9, we present the taxa identified as associated with biological features and dietary features by ZIQ-SIR or ZIQRank ($p < 0.05$ after FDR adjustment). Our method identifies more taxa with significant p-values, demonstrating higher power.

[Table 9 about here.]

References

Bosq, D. (1987). *Nonparametric Statistics for Stochastic Processes.* Springer New York, NY.

Cui, X., Härdle, W. K., and Zhu, L. (2011). The efm approach for single-index models.

He, X. and Shi, P. (1996). Bivariate tensor-product b-splines in a partly linear model. *Journal of Multivariate Analysis* **58,** 162–181.

Ling, W., Cheng, B., Wei, Y., Willey, J. Z., and Cheung, Y. K. (2022). Statistical inference in quantile regression for zero-inflated outcomes. *Statistica Sinica* **32,** 1411.

Ling, W., Zhang, W., Cheng, B., and Wei, Y. (2021). Zero-inflated quantile rank-score based test (ziqrank) with application to scrna-seq differential gene expression analysis. *The annals of applied statistics* **15,** 1673.

Ma, S. and He, X. (2016). Inference for single-index quantile regression models with profile optimization. *The Annals of Statistics* **44,**.

Ma, S., He, X., and Lemma, S. (2015). Supplement to “inference for single-index quantile regression models with profile optimization.”.

Wang, T., Ling, W., Plantinga, A. M., Wu, M. C., and Zhan, X. (2022). Testing microbiome association using integrated quantile regression models. *Bioinformatics* **38,** 419–425.

Zeileis, A., Kleiber, C., and Jackman, S. (2008). Regression models for count data in r. *Journal of statistical software* **27,** 1–25.

Zhan, X. and Wu, M. C. (2018). Reader reaction: A note on testing and estimation in marker-set association study using semiparametric quantile regression kernel machine. *Biometrics* **74,** 764–766.

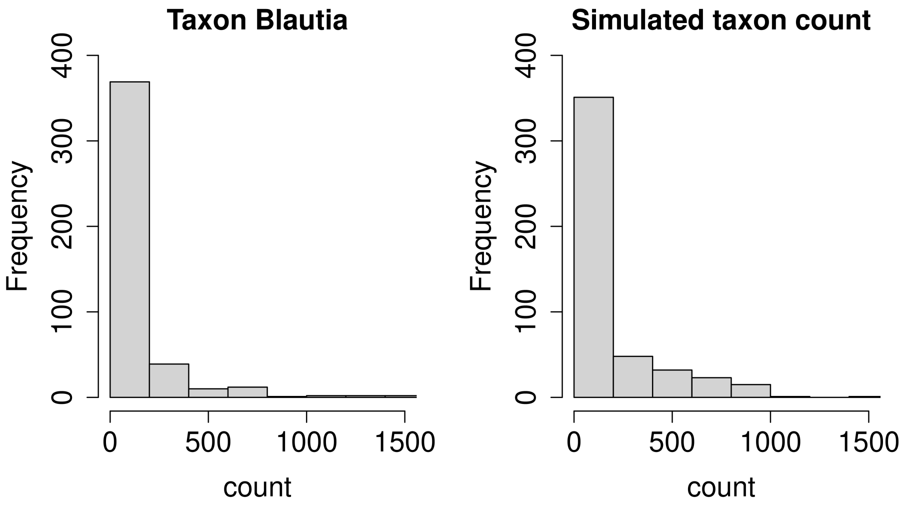

Figure S.1: The histogram of real taxon count and the taxon count simulated by Setting 2.

Table S.1: The correlation between some variables in real data.

	sex	waist	BMI	diastolic bp	systolic bp
sex	1.00	-0.07	-0.29	-0.12	-0.17
waist	-0.07	1.00	0.93	0.18	0.72
BMI	-0.29	0.93	1.00	0.48	0.86
diastolic bp	-0.12	0.18	0.48	1.00	0.70
systolic bp	-0.17	0.72	0.86	0.70	1.00

Table S.2: Type I error results with the significant threshold $\alpha = 0.01$; sample size $n = 500$.

		ZIQ-SIR	ZIQRank	Quantile Single-index	ZINB	ZIP
Setting 1	x_1	0.010	0.012	0.018	0.846	0.874
	x_2	0.012	0.018	0.022	0.774	0.766
	x_3	0.006	0.014	0.016	0.868	0.850
	x_4	0.008	0.012	0.024	0.860	0.874
	x_5	0.008	0.016	0.018	0.878	0.862
Setting 2	x_2	0.008	0.016	0.022	0.896	0.784
	x_3	0.012	0.026	0.030	0.854	0.836
	x_4	0.012	0.010	0.108	0.884	0.886
	x_5	0.006	0.010	0.180	0.840	0.858
	x_2, x_3	0.006	0.010	0.022	0.948	0.920
	x_4, x_5	0.008	0.014	0.008	0.970	0.992

Table S.3: Type I error results (without ZIP and ZINB) with the significant threshold $\alpha = 0.01$; sample size $n = 200$.

$\alpha = 0.01$		ZIQ-SIR	ZIQRank	Quantile Single-index
Setting 1	x_1	0.006	0.014	0.044
	x_2	0.012	0.020	0.054
	x_3	0.014	0.014	0.028
	x_4	0.016	0.008	0.046
	x_5	0.006	0.012	0.050
Setting 2	x_2	0.010	0.018	0.026
	x_3	0.008	0.010	0.042
	x_4	0.008	0.016	0.125
	x_5	0.006	0.018	0.022
	x_2, x_3	0.012	0.016	0.042
	x_4, x_5	0.010	0.012	0.054
$\alpha = 0.05$		ZIQ-SIR	ZIQRank	Quantile Single-index
Setting 1	x_1	0.050	0.070	0.132
	x_2	0.050	0.074	0.140
	x_3	0.054	0.056	0.100
	x_4	0.058	0.068	0.132
	x_5	0.046	0.056	0.134
Setting 2	x_2	0.056	0.062	0.148
	x_3	0.048	0.060	0.174
	x_4	0.040	0.066	0.450
	x_5	0.034	0.068	0.504
	x_2, x_3	0.048	0.066	0.120
	x_4, x_5	0.040	0.064	0.124

Table S.4: Type I error results (without ZIP and ZINB) with the significant threshold $\alpha = 0.01$; sample size $n = 2000$.

		ZIQ-SIR	ZIQRank	Quantile Single-index
Setting 1	x_1	0.004	0.018	0.010
	x_2	0.008	0.020	0.006
	x_3	0.008	0.016	0.014
	x_4	0.010	0.008	0.004
	x_5	0.010	0.020	0.016
Setting 2	x_2	0.016	0.022	0.012
	x_3	0.012	0.018	0.034
	x_4	0.010	0.010	0.098
	x_5	0.008	0.020	0.106
	x_2, x_3	0.008	0.018	0.018
	x_4, x_5	0.008	0.012	0.018

Table S.5: Power results (without ZINB and ZIP) for Setting 1 and 2; the significant threshold $\alpha = 0.01$; sample size $n = 2000$.

		ZIQ-SIR	ZIQRank	Quantile Single-index
Setting 1	x_1	0.744	0.704	0.050
	x_2	0.024	0.068	0.012
	x_3	0.842	0.826	0.052
	x_4	0.354	0.364	0.036
	x_5	0.294	0.274	0.038
Setting 2	x_2	0.052	0.080	0.030
	x_3	0.854	0.798	0.064
	x_4	0.100	0.096	0.140
	x_5	0.084	0.074	0.236
	x_2, x_3	0.766	0.738	0.020
	x_4, x_5	0.080	0.088	0.020

Table S.6: Type I error by Hurdle Poisson and Hurdle Negative Binomial models

		$n = 2000$		$n = 500$		$n = 200$	
		H-Poisson	H-NB	H-Poisson	H-NB	H-Poisson	H-NB
Setting 1	x_1	0.904	0.124	0.892	0.136	0.860	0.092
	x_2	0.972	0.126	0.968	0.088	0.988	0.074
	x_3	0.950	0.094	0.948	0.082	0.970	0.064
	x_4	0.970	0.332	0.950	0.268	0.984	0.156
	x_5	0.954	0.234	0.940	0.184	0.968	0.148
Setting 2	x_2	0.980	0.090	0.990	0.054	0.984	0.062
	x_3	0.996	0.122	0.968	0.074	0.960	0.070
	x_4	0.982	0.234	0.964	0.104	0.948	0.064
	x_5	0.978	0.180	0.994	0.096	0.986	0.054
	x_2, x_3	0.996	0.422	0.994	0.336	0.998	0.082
	x_4, x_5	0.998	0.106	0.998	0.088	0.998	0.058

Table S.7: Type I error result with the significant threshold $\alpha = 0.05$ for count data.

$n = 500$		ZIQ-SIR	ZIQRank	Quantile Single-index
Setting 1	x_1	0.046	0.070	0.082
	x_2	0.042	0.068	0.062
	x_3	0.042	0.052	0.074
	x_4	0.050	0.064	0.082
	x_5	0.038	0.062	0.090
Setting 2	x_2	0.046	0.060	0.098
	x_3	0.040	0.056	0.110
	x_4	0.042	0.070	0.334
	x_5	0.050	0.062	0.370
	x_2, x_3	0.052	0.074	0.066
	x_4, x_5	0.052	0.062	0.094
$n = 2000$		ZIQ-SIR	ZIQRank	Quantile Single-index
Setting 1	x_1	0.052	0.048	0.076
	x_2	0.038	0.054	0.068
	x_3	0.056	0.052	0.058
	x_4	0.054	0.058	0.058
	x_5	0.052	0.050	0.072
Setting 2	x_2	0.048	0.084	0.072
	x_3	0.050	0.050	0.070
	x_4	0.056	0.062	0.434
	x_5	0.054	0.058	0.268
	x_2, x_3	0.046	0.056	0.042
	x_4, x_5	0.046	0.050	0.068

Table S.8: Power result with the significant threshold $\alpha = 0.05$ for count data.

$n = 500$		ZIQ-SIR	ZIQRank	Quantile Single-index
Setting 1	x_1	0.444	0.456	0.134
	x_2	0.082	0.080	0.072
	x_3	0.566	0.550	0.092
	x_4	0.278	0.256	0.106
	x_5	0.250	0.240	0.104
Setting 2	x_2	0.072	0.082	0.104
	x_3	0.310	0.274	0.094
	x_4	0.070	0.068	0.332
	x_5	0.088	0.074	0.424
	x_2, x_3	0.284	0.222	0.108
	x_4, x_5	0.108	0.098	0.066
$n = 2000$		ZIQ-SIR	ZIQRank	Quantile Single-index
Setting 1	x_1	0.998	0.990	0.386
	x_2	0.136	0.144	0.108
	x_3	0.998	0.998	0.366
	x_4	0.876	0.852	0.180
	x_5	0.844	0.796	0.312
Setting 2	x_2	0.156	0.178	0.076
	x_3	0.906	0.880	0.192
	x_4	0.258	0.238	0.294
	x_5	0.210	0.176	0.462
	x_2, x_3	0.896	0.872	0.126
	x_4, x_5	0.164	0.146	0.106

Table S.9: The FDR adjusted p-values by ZIQ-SIR and ZIQRank methods.

Taxa associated with the **biological features**	ZIQ-SIR	ZIQRank
Peptococcaceae-unspecified	**0.030***	0.408
Rhizobiales-unspecified-unspecified	**0.030***	0.331
Actinomycetales-unspecified-unspecified	**0.030***	**0.033***
Taxa associated with the **dietary features**	ZIQ-SIR	ZIQRank
Peptococcaceae-unspecified	**1.57e-13***	**3.15e-13***
Peptostreptococcus	**1.21e-13***	0.697
