# Supplementary material for: A semiparametric quantile regression rank score test for zero-inflated data
Source: Biometrics. 2025 May 5;81(2):ujaf050. doi: 10.1093/biomtc/ujaf050 (PMC12050976; doi:10.1093/biomtc/ujaf050)
Supplement: ujaf050_Supplemental_Files — Web Appendices, tables, figures, and code referenced in Sections 2, 3, and 4 are available with this paper at the Biometrics website on Oxford Academic. [file ujaf050_supplemental_files.zip › ZIQ-SIR- code.zip!/ZIQ-SIR-main/ZIQ-SIR.pdf]

Package ‘ZIQSIR’

February 20, 2025

Type Package

Title ZIQ-SIR package

Version 0.1.0

Author Zirui Wang, Tianying Wang

Maintainer Zirui Wang <wzr23@mails.tsinghua.edu.cn>,
Tianying Wang <Tianying.Wang@colostate.edu>

Description
We provide a novel semiparametric single-index method to test the relationship between the covariate(s) of interest and zero-inflated data. By inputting the response variable, explanatory variable(s), the indices of the variables to be tested, and the method to be used, we can test the relationship between the variables of interest and the zero-inflated response variable.

License GPL-2

Encoding UTF-8

LazyData true

Depends R (>= 3.5.0),

Imports MASS,
lme4,
splines2,
PearsonDS,
quantreg

RoxygenNote 7.3.2

R topics documented:

ZIQSIR 1

Index **5**

`ZIQSIR` *Provides a novel tool to obtain the p-value for testing associations for zero-inflated response.*

Description

Provides a novel tool to obtain the p-value for testing associations for zero-inflated response.

Usage

```
ZIQSIR(
  y,
  X,
  taus = c(0.1, 0.25, 0.5, 0.75, 0.9),
  m = 3,
  test_num,
  method = "Chi"
)
```

Arguments

y	n*1 vector, the observed outcome for n samples.
X	n*p matrix, the observed p covariates for n samples.
taus	k*1 vector, a grid of quantile levels; e.g., 0.5 for the median, 0.75 for the 3rd quartile; default is c(0.1, 0.25, 0.5, 0.75, 0.9).
m	numeric variable, the order of B-spline function; default is 3.
test_num	a vector, representing the test corresponds to which covariate(s) in X.
method	different method for calculating p-value: 'large' for large sample cases, where we use the asymptotic distribution for hypothesis testing; 'small' for small sample cases, where we use the Pearson Type III distribution to approximate the null distribution.

Details

- Please choose 'large' or 'small' for `method`, no other options.
- `taus` must be a subset or equal to the grid used to produce `input`.

Value

A p-value for testing the association between the covariate(s) of interest and the zero-inflated response.

Examples

```
# example code
#### demo 1:
# small sample size
# using Pearson Type III method
# alternative distribution
# sample size
set.seed(10001)
n = 500
# the probability of Y>0 given covariate x
p = function(x1,x2,x3,x4,x5,gam0=-0.4,gam1=-0.480,gam2=-0.022,gam3=0.021,gam4 = 0.015,gam5 = -0.009){
  lc = gam0 + gam1*x1 + gam2*x2 + gam3*x3 + gam4*x4 + gam5*x5
  exp(lc)/(1+exp(lc))
}
# beta_tau
bet1 = function(x){(0.3*sqrt(x)-x)*2}
bet2 = function(x){x^2*2.2}
bet3 = function(x){
```

```
  (x^2-0.5*x+0.6)*2/3
}
bet5 = function(x){-(0.3*x^2-x)*2}
bet4 = function(x){-sin(x*2*pi)*0.1}
bet0 = function(x){-147.7*x-50*x^2-20}
bet = function(x,u){x^4*u*10^(-5)/6+x^2*u*0.2/3}
# G_tau function
func <- function(x, tau)
{
 return(bet(x %*% rbind(bet1(tau),bet2(tau),bet3(tau),bet4(tau),bet5(tau))+bet0(tau),tau))
}
# the given covariate x
X1 = c(0,1) # sex
X2 = qnorm(0.5,28,2) # bmi
X3 = qnorm(0.5,92.5,13) # waist
X4 = qnorm(0.5,80,12) # diastolic_bp
X5 = qnorm(0.5,124,18.5) # systolic_bp
X0 =cbind(c(rep(X1[1], 1), rep(X1[2], 1)),  rep(X2, 2), rep(X3, 2), rep(X4, 2), rep(X5,2))
# given samples
x1 = rbinom(n,1,0.5) # Medicament use
x2 = rnorm(n,28,2) # bmi
x3 = rnorm(n,92.5,13) # waist
x4 = rnorm(n,80,12) # diastolic_bp
x5 = rnorm(n,124,18.5) # systolic_bp
x0 = rep(1,n)
X = cbind(x0,x1,x2,x3,x4,x5)
u = runif(n)
b = rbinom(n,1,p(x1,x2,x3,x4,x5))
w = bet(bet1(u)*x1+bet2(u)*x2+bet3(u)*x3+bet4(u)*x4+bet5(u)*x5+bet0(u),u)
y = b*w

ZIQSIR(y,X,m = 3,test_num = 4,method = "small")

### demo 2
# simulation results under large sample size
# using Chi-square method
# under null hypothesis
# sample size
n = 2000

# the probability of Y>0 given covariate x
p = function(x1,x2,x3,x4,x5,x6,
         gam0=2.32,gam1=-0.06,gam2=-0.03*0,gam3=-0.010*0,gam4 = -0.005,gam5 = 0.0005,gam6 = -0.030){
  lc = gam0 + gam1*x1 + gam2*x2 + gam3*x3 + gam4*x4 + gam5*x5 + gam6*x6
  exp(lc)/(1+exp(lc))
}

# beta_tau
bet1 = function(x){5*x^2+1}
bet2 = function(x){(0.1*sin(x*2*pi)+0.05)*0}
bet3 = function(x){
  ( 0.05*(x-0.5)^2+0.04)*0
}
bet4 = function(x){(0.1*sqrt(x)+2.9*x)*0.05}
bet5 = function(x){(0.4*(x-1)^2)*0.1}
bet6 = function(x){((x-0.6)*(x-1.1))*1.1}
bet0 = function(x){62.9*x^2+33.4*x}
```

```
bet = function(x,u){x^4*u*10^(-4)*0.4+x^3*u*10^{-3}*0.1}

# G_tau function
func <- function(x, tau)
{
  return(bet(x %*% rbind(bet1(tau),bet2(tau),bet3(tau),bet4(tau),bet5(tau),bet6(u))+bet0(tau),tau))
}

# given samples
x1 = rbinom(n,1,0.5) # Gender
x2 = rnorm(n,28,2) # bmi
x3 = 2*x2+rnorm(n,36.5,9) # waist
x4 = rnorm(n,80,12) # diastolic_bp
x5 = x4*1.3+rnorm(n,20,7.75) # systolic_bp
x6 = sample(1:4, n, replace = TRUE, prob = c(0.25,0.025,0.07,0.625))
x0 = rep(1,n)
u = runif(n)
b = rbinom(n,1,p(x1,x2,x3,x4,x5,x6))
w = bet(bet1(u)*x1+bet2(u)*x2+bet3(u)*x3+bet4(u)*x4+bet5(u)*x5+bet6(u)*x6+bet0(u),u)
y = b*w
X = cbind(x1,x2,x3,x4,x5,x6)

ZIQSIR(y,X,m = 3,test_num = c(2,3),method = "large")
```

Index

ZIQSIR, 1
