# Supplementary material for: A semiparametric quantile regression rank score test for zero-inflated data
Source: Biometrics. 2025 May 5;81(2):ujaf050. doi: 10.1093/biomtc/ujaf050 (PMC12050976; doi:10.1093/biomtc/ujaf050)
Supplement: ujaf050_Supplemental_Files — Web Appendices, tables, figures, and code referenced in Sections 2, 3, and 4 are available with this paper at the Biometrics website on Oxford Academic. [file ujaf050_supplemental_files.zip › ZIQ-SIR- code.zip!/ZIQ-SIR-main/ZIQSIR_0.1.0.tar.gz!/ZIQSIR/README.html]

README


# Powerful and robust non-parametric association testing for zero-inflated data via a zero-inflated quantile approach (ZIQ-SIR)

## Introduction

The **ZIQ-SIR** R package is designed to provide
advanced statistical tools for association testing for zero-inflated
data. This package includes one main function:

- `Combination`: A function for testing association for
  zero-inflated data and covariates of interest.

This document provides an overview of the package, how to install it,
and examples of how to use these functions.

## Usage

## Installation

To install the **ZIQSI** package from GitHub, you can
use the following code:

`{r} # If devtools is not already installed, install it first # install.packages("devtools") # Install the ZIQSI package from GitHub devtools::install_github("https://github.com/tianyingw/ZIQ-SIR")`

## License

This R file is free and open source software, licensed under GPL
(>=2)

## Author

Zirui Wang (wzr23@mails.tsinghua.edu.cn) and Tianying Wang (Tianying.Wang@colostate.edu)
